# Dried blood spot specimens for SARS-CoV-2 antibody testing: A multi-site, multi-assay comparison

**DOI:** 10.1371/journal.pone.0261003

**Published:** 2021-12-07

**Authors:** François Cholette, Christine Mesa, Angela Harris, Hannah Ellis, Karla Cachero, Philip Lacap, Yannick Galipeau, Marc-André Langlois, Anne-Claude Gingras, Cedric P. Yansouni, Jesse Papenburg, Matthew P. Cheng, Pranesh Chakraborty, Derek R. Stein, Paul Van Caeseele, Sofia Bartlett, Mel Krajden, David Goldfarb, Allison McGeer, Carla Osiowy, Catherine Hankins, Bruce Mazer, Michael Drebot, John Kim, on behalf of the COVID-19 Immunity Task Force (CITF) working group

**Affiliations:** 1 National Microbiology Laboratory, Public Health Agency of Canada, Winnipeg, Canada; 2 Department of Medical Microbiology and Infectious Diseases, University of Manitoba, Winnipeg, Canada; 3 Department of Biochemistry, Microbiology, and Immunology, University of Ottawa, Ottawa, Canada; 4 The Centre for Infection, Immunity, and Inflammation (CI3), University of Ottawa, Ottawa, Canada; 5 Department of Molecular Genetics, University of Toronto, Toronto, Canada; 6 Lunenfeld-Tanenbaum Research Institute at Mount Sinai Hospital, Sinai Health System, Toronto, Canada; 7 McGill Interdisciplinary Initiative in Infection and Immunity, McGill University, Montréal, Canada; 8 Division of Microbiology, Department of Clinical Laboratory Medicine, Optilab Montréal–McGill University Health Centre, Montréal, Canada; 9 Newborn Screening Ontario, Children’s Hospital of Eastern Ontario, Ottawa, Canada; 10 Cadham Provincial Laboratory, Manitoba Health, Winnipeg, Canada; 11 Department of Pediatrics & Child Health, University of Manitoba, Winnipeg, Canada; 12 COVID-19 Immunity Task Force, Montréal, Canada; 13 British Columbia Centre for Disease Control, Vancouver, Canada; 14 Department of Pathology and Laboratory Medicine, University of British Columbia, Vancouver, Canada; 15 Division of Infectious Diseases, University of Toronto, Toronto, Canada; 16 Department of Epidemiology, Biostatistics, and Occupational Health, McGill University, Montréal, Canada; 17 Department of Pediatrics, McGill University, Montréal, Canada; Qatar University, QATAR

## Abstract

The true severity of infection due to COVID-19 is under-represented because it is based on only those who are tested. Although nucleic acid amplifications tests (NAAT) are the gold standard for COVID-19 diagnostic testing, serological assays provide better population-level SARS-CoV-2 prevalence estimates. Implementing large sero-surveys present several logistical challenges within Canada due its unique geography including rural and remote communities. Dried blood spot (DBS) sampling is a practical solution but comparative performance data on SARS-CoV-2 serological tests using DBS is currently lacking. Here we present test performance data from a well-characterized SARS-CoV-2 DBS panel sent to laboratories across Canada representing 10 commercial and 2 in-house developed tests for SARS-CoV-2 antibodies. Three commercial assays identified all positive and negative DBS correctly corresponding to a sensitivity, specificity, positive predictive value, and negative predictive value of 100% (95% CI = 72.2, 100). Two in-house assays also performed equally well. In contrast, several commercial assays could not achieve a sensitivity greater than 40% or a negative predictive value greater than 60%. Our findings represent the foundation for future validation studies on DBS specimens that will play a central role in strengthening Canada’s public health policy in response to COVID-19.

## Introduction

According to the latest estimates from John Hopkins University (last accessed on 9 September 2021), severe acute respiratory syndrome coronavirus (SARS-CoV2), the etiological agent of coronavirus disease 2019 (COVID-19) [1], is responsible for over 220 million confirmed cases, including over 4.5 million deaths globally [2]. While nucleic acid amplification tests (NAAT) on respiratory samples remain the gold standard for COVID-19 diagnostic testing [3], they cannot provide reliable prevalence and incidence estimates at the population level given the narrow window for reliable results (<14 days post-symptom onset) [4], global supply shortages [5], and limited access due to symptom-based testing prioritization [6]. On the other hand, serological assays may provide more reliable population-level SARS-CoV-2 prevalence estimates to better inform ongoing public health responses [7]. Serological testing is also vitally important for monitoring both individual and population-level humoral immune responses to COVID-19 vaccination [8].

Several commercial serological assays rapidly became available through measures such as the US Food and Drug Administration’s (FDA) Emergency Use Authorization programme [9]. These assays have been primarily designed for the detection of SARS-CoV-2 antibodies in serum, plasma, or whole blood [10–13]. Implementing large-scale integrated biological-behavioural surveys poses a significant challenge since phlebotomy requires highly trained personnel when most health care professionals have been re-deployed to assist in the COVID-19 response [14]. Furthermore, reaching rural and remote communities adds to the complexity of providing reliable and timely testing for several reasons including lack of trained personnel to collect biological specimens, limited access to laboratory facilities, difficulties in maintaining the cold chain, and unreliable specimen transportation even within the Canadian context. The implementation of SARS-CoV-2 point-of-care (POC) testing [15–18] could alleviate some of these challenges but may not be appropriate in all settings. Rural and remote communities tend to be small and have close-knit social networks thereby making confidential or anonymous SARS-CoV-2 POC testing difficult. Thus, a practical solution is required to be able to achieve large scale sampling and circumvent these problematic issues. Dried blood spot (DBS) collection may be a practical solution owing to this method’s simplicity.

DBS are prepared by placing a few drops of blood on a card made of filter paper. A finger prick is performed using standard spring-loaded lancets that do not require specialised training to utilize. Home-self collection is also a viable option that is already playing a significant role in SARS-CoV-2 sero-surveillance [19,20]. Once the DBS cards are dry, they can be stored and transported at ambient temperature to centralized laboratories through regular mail services without any adverse effect on downstream testing [21]. However, comparative performance data on SARS-CoV-2 serological assays using DBS specimens are limited. Here we present test performance data from a SARS-CoV-2 DBS panel sent to various public health and academic laboratories across Canada representing 10 commercial and 2 in-house developed assays for SARS-CoV-2 antibodies. As part of the COVID-19 Immunity Task Force (CITF, www.covid19immunitytaskforce.ca/), our goal was to provide preliminary performance data on each test to guide future large-scale validations.

## Results

### Assay performance

**Fig 1:**
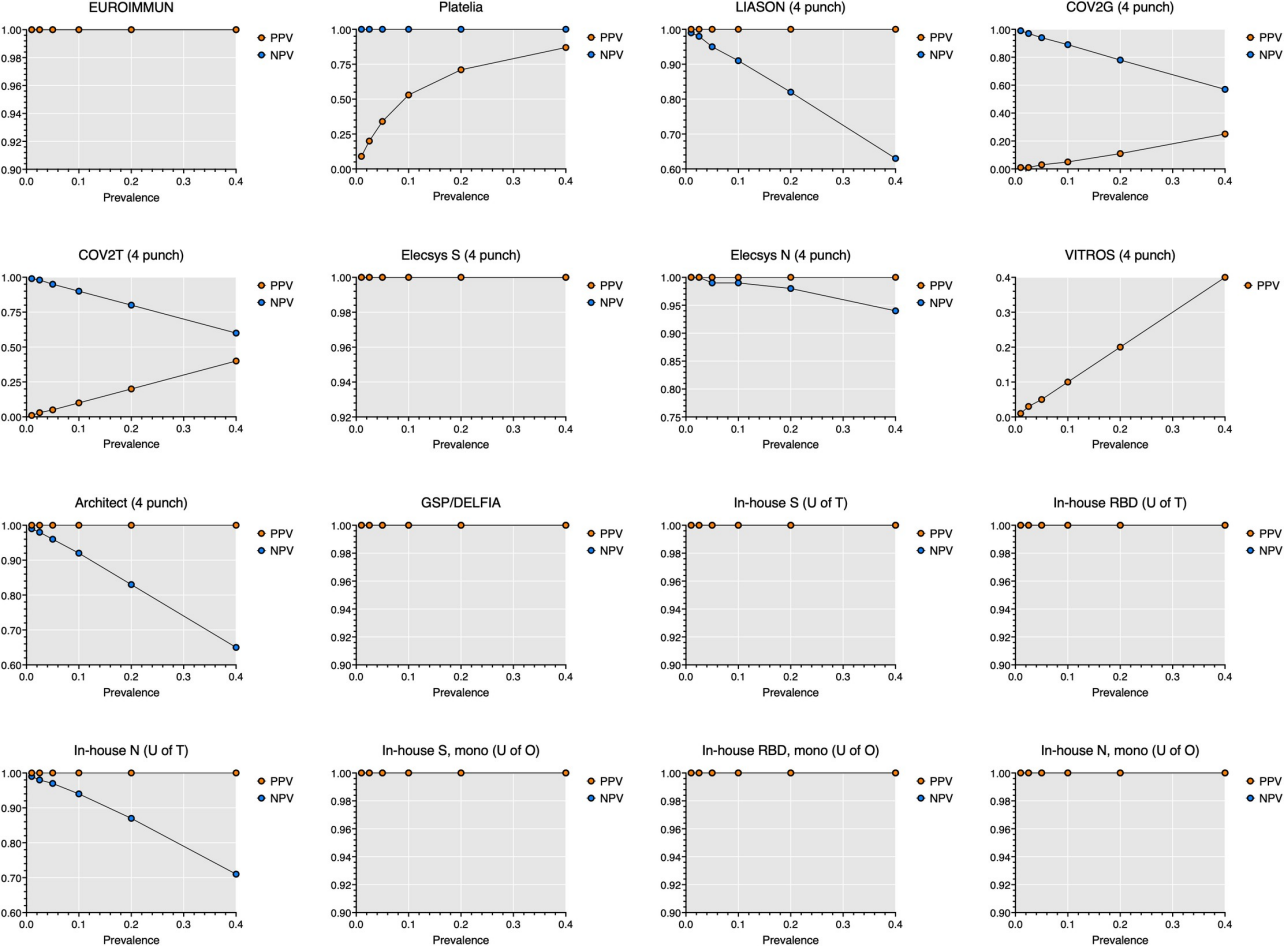
Positive predictive values (PPV) and negative predictive values (NPV) by prevalence for each commercial and in-house assay on dried blood spot specimens. PPV and NPV is presented for *n =* 10 SARS-CoV-2 antibody negative DBS specimens and *n* = 10 SARS-CoV-2 antibody positive DBS specimens. One 6 mm (1/4 inch) punch was used for the EUROIMMUN assay and two 6 mm (1/4 inch) punches were used for the Platelia and in-house assays. Data from four 6 mm (1/4 inch) punches is presented for all other assays. EUROIMMUN = Anti-SARS-CoV-2 ELISA assay (EUROIMMUN, Lübeck, Germany). Platelia = SARS-CoV-2 assay (Bio-Rad, Hercules, California). LIASON = SARS-CoV-2 assay (DiaSorin, Saluggia, Italy). COV2G = SARS-CoV-2 COV2G assay (Siemens, Erlangen, Germany). COV2T = SARS-CoV-2 COV2T assay (Siemens). Elecsys S = Quantitative Anti-SARS-CoV-2 assay (Elecsys spike; Roche, Basel, Switzerland). Elecsys N = Anti-SARS-CoV-2 assay (Elecsys nucleocapsid; Roche). VITROS = Anti-SARS-CoV-2 assay (Ortho Clinical Diagnostics, Raritan, New Jersey). Architect = SARS-CoV-2 assay (Abbott, Mississauga, Canada). GSP/DELFIA = Anti-SARS-CoV-2 assay (PerkinElmer, Waltham, Massachusetts). In-house S (U of T) = In-house spike assay (University of Toronto). In-house RBD (U of T) = In-house RBD assay (University of Toronto). In-house N (U of T) = In-house nucleocapsid assay (University of Toronto). In-house S, mono (U of O) = In-house monoclonal spike assay (University of Ottawa). In-house RBD, mono (U of O) = In-house monoclonal RBD assay (University of Ottawa). In-house N, mono (U of O) = In-house monoclonal nucleocapsid assay (University of Ottawa). The VITROS assay could not achieve a specificity greater than 0% therefore, only PPV is shown.

**Fig 2:**
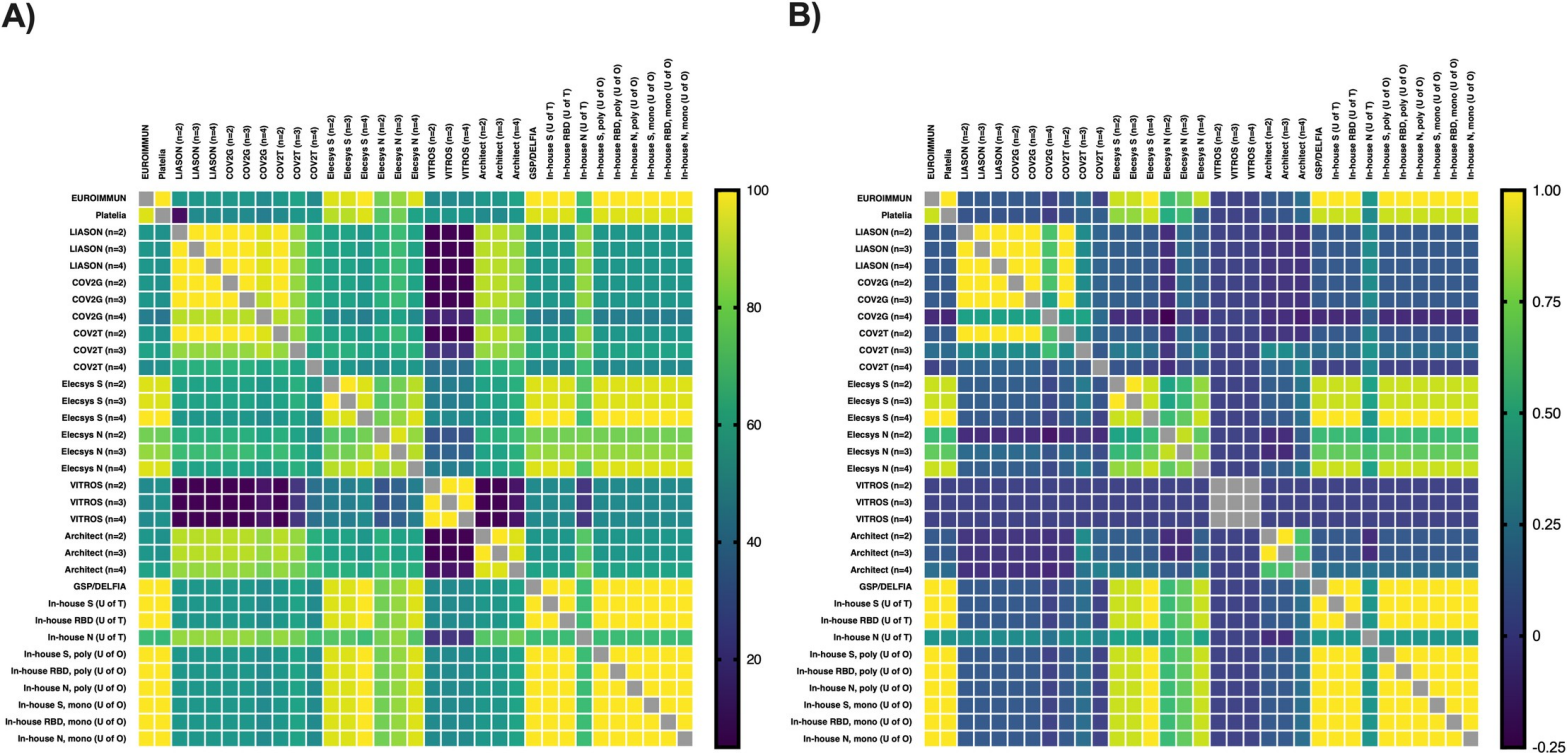
Inter-assay concordance (A) and Cohen’s kappa coefficient (B) for SARS-CoV-2 antibody testing on dried blood spot specimens. Concordance (%) and Cohen’s kappa coefficient are presented for n = 10 SARS-CoV-2 antibody negative DBS specimens and n = 10 SARS-CoV-2 antibody positive DBS specimens. One 6 mm (1/4 inch) punch was used for the EUROIMMUN assay and two 6 mm (1/4 inch) punches were used for the Platelia and in-house assays. EUROIMMUN = Anti-SARS-CoV-2 ELISA assay (EUROIMMUN, Lübeck, Germany). Platelia = SARS-CoV-2 assay (Bio-Rad, Hercules, California). LIASON = SARS-CoV-2 assay (DiaSorin, Saluggia, Italy). COV2G = SARS-CoV-2 COV2G assay (Siemens, Erlangen, Germany). COV2T = SARS-CoV-2 COV2T assay (Siemens). Elecsys S = Quantitative Anti-SARS-CoV-2 assay (Elecsys spike; Roche, Basel, Switzerland). Elecsys N = Anti-SARS-CoV-2 assay (Elecsys nucleocapsid; Roche). VITROS = Anti-SARS-CoV-2 assay (Ortho Clinical Diagnostics, Raritan, New Jersey). Architect = SARS-CoV-2 assay (Abbott, Mississauga, Canada). GSP/DELFIA = Anti-SARS-CoV-2 assay (PerkinElmer, Waltham, Massachusetts). In-house S (U of T) = In-house spike assay (University of Toronto). In-house RBD (U of T) = In-house RBD assay (University of Toronto). In-house N (U of T) = In-house nucleocapsid assay (University of Toronto). In-house S, mono (U of O) = In- house monoclonal spike assay (University of Ottawa). In-house RBD, mono (U of O) = In-house monoclonal RBD assay (University of Ottawa). In-house N, mono (U of O) = In-house monoclonal nucleocapsid assay (University of Ottawa).

Sensitivity, specificity, positive predictive values (PPV), and negative predictive values (NPV) were assessed for 10 commercial and 2 in-house assays on 10 known negative plasma samples and 10 plasma samples from COVID-19 patients (Table 1) contrived as DBS. Nearly one third of all commercial assays (EUROIMMUN, Elecsys spike, and GSP/DELFIA) identified all positive and negative DBS correctly corresponding to a sensitivity, specificity, PPV, and NPV of 100% (95% CI = 72.2, 100) even when using a wide range of prevalence estimates (Figs 1 and S1). The Elecsys spike and GSP/DELFIA showed almost perfect agreement with EUROIMMUN as well as with one another (Tables 1 and S1, Fig 2).

**Table 1:** Assay performance for SARS-CoV-2 antibody testing on dried blood spot specimens (DBS).

Assay	DBS punches (*n* =)	TP (*n* =)	FN (*n* =)	TN(*n* =)	FP (*n* =)	Eq (*n* =)	Se (95% CI [LL, UL])	Sp (95% CI [LL, UL])	PPV (95% CI [LL, UL])	NPV (95% CI [LL, UL])	Kappa^a^ (95% CI [LL, UL])
**EUROIMMUN**	2	10	0	10	0	0	100% (72.2, 100)	100% (72.2, 100)	100% (72.2, 100)	100% (72.2, 100)	Reference
**Platelia**	2	10	0	9	0	1	100% (72.2, 100)	90% (59.6, 99.5)	90.9% (62.3, 99.5)	100% (70.1, 100)	0.95 (0.86, 1.00)
**LIASON**	2	1	9	10	0	0	10% (0.5, 40.4)	100% (72.2, 100)	100% (5.1, 100)	52.6% (31.7, 72.7)	0.45 (0.20, 0.70)
**LIASON**	3	1	9	10	0	0	10% (0.5, 40.4)	100% (72.2, 100)	100% (5.1, 100)	52.6% (31.7, 72.7)	0.45 (0.20, 0.70)
**LIASON**	4	1	9	10	0	0	10% (0.5, 40.4)	100% (72.2, 100)	100% (5.1, 100)	52.6% (31.7, 72.7)	0.45 (0.20, 0.70)
**COV2G**	2	1	9	10	0	0	10% (0.5, 40.4)	100% (72.2, 100)	100% (5.1, 100)	53% (32, 73)	0.05 (-0.26, 0.36)
**COV2G**	3	1	9	10	0	0	10% (0.5, 40.4)	100% (72.2, 100)	100% (5.1, 100)	53% (32, 73)	0.05 (-0.26, 0.36)
**COV2G**	4	1	9	8	2	0	10% (0.5, 40.4)	80% (49, 96.4)	33.3% (1.7, 88.2)	47.1% (26.2, 69)	0.15 (-0.16, 0.46)
**COV2T**	2	1	9	10	0	0	10% (0.5, 40.4)	100% (72.2, 100)	100% (5.1, 100)	52.6% (31.7, 72.7)	0.55 (0.32, 0.78)
**COV2T**	3	3	7	9	1	0	30% (10.8, 60.3)	90% (59.6, 99.5)	75% (30.1, 98.7)	56.3% (33.2, 76.9)	0.60 (0.36, 0.84)
**COV2T**	4	4	6	6	4	0	40% (16.8, 68.7)	60% (31.3, 83.2)	50% (21.5, 78.5)	50% (25.4, 74.6)	0.50 (0.23, 0.77)
**Elecsys S**	2	9	1	0	10	0	90% (59.6, 99.5)	100% (72.2, 100)	100% (70.1, 100)	90.9% (62.3, 99.5)	0.45 (0.203, 0.70)
**Elecsys S**	3	9	1	0	10	0	90% (59.6, 99.5)	100% (72.2, 100)	100% (70.1, 100)	90.9% (62.3, 99.5)	0.45 (0.203, 0.70)
**Elecsys S**	4	10	0	10	0	0	100% (72.2, 100)	100% (72.2, 100)	100% (72.2, 100)	100% (72.2, 100)	1.00 (1.00, 1.00)
**Elecsys N**	2	6	4	10	0	0	60% (31.3, 83.2)	100% (72.2, 100)	100% (72.2, 100)	71.4% (45.4, 88.3)	0.80 (0.62, 0.98)
**Elecsys N**	3	7	3	10	0	0	70% (39.7, 89.2)	100% (72.2, 100)	100% (39.7, 89.2)	76.9% (49.7, 91.8)	0.85 (0.69, 1.00)
**Elecsys N**	4	9	1	10	0	0	90% (59.6, 99.5)	100% (72.2, 100)	100% (70.1, 100)	90.9% (62.3, 99.5)	0.95 (0.85, 1.00)
**VITROS**	2	10	0	0	10	0	100%(72.2–100)	0% (0, 27.8)	50% (29.9, 70.1)	N/A	0.50 (0.27, 0.73)
**VITROS**	3	10	0	0	10	0	100% (72.2, 100)	0% (0, 27.8)	50% (29.9, 70.1)	N/A	0.50 (0.27, 0.73)
**VITROS**	4	10	0	0	10	0	100% (72.2, 100)	0% (0, 27.8)	50% (29.9, 70.1)	N/A	0.50 (0.27, 0.73)
**Architect**	2	1	9	0	10	0	10% (0.5, 40.4)	100% (72.2, 100)	100% (5.1, 100)	52.6%(31.7, 72.7)	0.05 (-0.26, 0.36)
**Architect**	3	2	8	0	10	0	20% (3.6, 51)	100% (72.2, 100)	100% (17.8, 100)	55.6% (33.7, 75.4)	0.10 (-0.21, 0.41)
**Architect**	4	2	8	0	10	0	20% (3.6, 51)	100% (72.2, 100)	100% (17.8, 100)	55.6% (33.7, 75.4)	0.10 (-0.21, 0.41)
**GSP/DELFIA**	2	10	0	10	0	0	100% (72.2, 100)	100% (72.2, 100)	100% (72.2, 100)	100% (72.2, 100)	1.00 (1.00, 1.00)
**In-house S (U of T)**	2	10	0	10	0	0	100% (72.2, 100)	100% (72.2, 100)	100% (72.2, 100)	100% (72.2, 100)	1.00 (1.00, 1.00)
**In-house RBD (U of T)**	2	10	0	10	0	0	100% (72.2, 100)	100% (72.2, 100)	100% (72.2, 100)	100% (72.2, 100)	1.00 (1.00, 1.00)
**In-house N (U of T)**	2	4	6	10	0	0	40% (16.8, 68.7)	100% (72.2, 100)	100% (51.0, 100)	62.5% (38.6, 81.5)	0.70 (0.49, 0.91)
**In-house S, polyclonal (U of O)**	2	10	0	10	0	0	100% (72.2, 100)	100% (72.2, 100)	100% (72.2, 100)	100% (72.2, 100)	1.00 (1.00, 1.00)
**In-house RBD, polyclonal (U of O)**	2	10	0	10	0	0	100% (72.2, 100)	100% (72.2, 100)	100% (72.2, 100)	100% (72.2, 100)	1.00 (1.00, 1.00)
**In-house N, polyclonal (U of O)**	2	10	0	10	0	0	100% (72.2, 100)	100% (72.2, 100)	100% (72.2, 100)	100% (72.2, 100)	1.00 (1.00, 1.00)
**In-house S, monoclonal (U of O)**	2	10	0	10	0	0	100% (72.2, 100)	100% (72.2, 100)	100% (72.2, 100)	100% (72.2, 100)	1.00 (1.00, 1.00)
**In-house RBD, monoclonal (U of O)**	2	10	0	10	0	0	100% (72.2, 100)	100% (72.2, 100)	100% (72.2, 100)	100% (72.2, 100)	1.00 (1.00, 1.00)
**In-house N, monoclonal (U of O)**	2	10	0	10	0	0	100% (72.2, 100)	100% (72.2, 100)	100% (72.2, 100)	100% (72.2, 100)	1.00 (1.00, 1.00)

DBS punches: 6 mm (1/4 inch) dried blood spot punch; TP: True positive; FN: False negative; TN: True negative; FP: False positive; Eq: Equivocal; Se: Sensitivity; Sp: Specificity; PPV: Positive predictive value; NPV: Negative predictive value; 95% CI (LL, UL): 95% confidence intervals (lower limit, upper limit); S: Spike; RBD: Receptor binding domain; N: Nucleocapsid; U of T: University of Toronto; U of O: University of Ottawa.

^a^Kappa statistics interpreted as follows: <0 = no agreement, 0–0.20 = slight agreement, 0.21–0.40 = fair agreement, 0.41–0.60 = moderate agreement, 0.61–0.80 = substantial agreement, and 0.81–1.00 = almost perfect agreement.

The panel consisted of 10 unique SARS-CoV-2 antibody positive DBS cards contrived from patient samples and 10 unique SARS-CoV-2 negative DBS cards spotted directly from EDTA whole blood.

The Platelia assay achieved a sensitivity, specificity, PPV, and NPV of 100% (95% CI = 72.2, 100), 90% (95% CI = 59.6, 99.5), 90.9% (95% CI = 62.3, 99.5), and 100% (95% CI = 70.1, 100) respectively due to one equivocal result from a negative DBS sample. As a result, it appears that the Platelia assay can only achieve a PPV ≥90% in high prevalence settings (Fig 1). The Platelia assay showed almost perfect agreement with EUROIMMUN (Table 1) and other high performing commercial assays (S1 Table, Fig 2). The Elecsys nucleocapsid assay achieved a sensitivity, specificity, PPV, and NPV of 90% (95% CI = 59.6, 99.5), 100% (95% CI = 72.2, 100), 100% (95% CI = 70.1, 100), and 90.9% (95% CI = 62.3, 99.5) respectively after increasing the sample input from 2 to 4 DBS punches. Increasing the sample input also significantly reduces the number of false negatives in high prevalence settings (S1 Fig) and improves agreement with other commercial assays such as EUROIMMUN, Platelia, GSP/DELFIA, and Elecsys spike (Tables 1 and S1, Fig 2).

Performance varied for the remaining commercial assays. The LIASON, Architect, and COV2G assays achieved a specificity and PPV of 100% (95% CI = 72.2, 100) and 100% (95% CI = 5.1, 100) respectively but could not achieve a sensitivity greater than 20% (95% CI = 3.6, 51) or an NPV greater than 55.6% (95% CI = 33.7, 75.4) despite increasing the sample input from 2 to 4 DBS punches. The COV2T assay achieved a sensitivity, specificity, PPV, and NPV of 40% (95% CI = 16.8, 68.7), 60% (95% CI = 31.3, 83.2), 50% (95% CI = 21.5, 78.5), and 50% (95% CI = 25.4, 74.6) respectively after increasing the sample input from 2 to 4 DBS punches. Similarly, the VITROS assay achieved a sensitivity and PPV of 100% (95% CI = 72.2, 100) and 50% (95% CI = 29.9, 70.1) respectively but could not achieve a specificity greater than 0% despite increasing sample input. This would suggest that the LIASON and Architect assays would yield a high number of false negative results in high prevalence settings (≥5.0%) while the COV2G and COV2T assays would yield a high number of false negative and false positive results in low to high prevalence settings (Figs 1 and S1). The VITROS assay is likely not to be useful under any prevalence setting since all DBS samples produced positive results (Table 1). As expected, these commercial assays had no agreement to moderate agreement with EUROIMMUN (Table 1) and the higher performing assays Platelia, Elecsys spike, Elecsys nucleocapsid, and GSP/DELFIA (S1 Table, Fig 2).

The in-house assays from the University of Ottawa and University of Toronto performed well and successfully identified all positive and negative DBS correctly corresponding to a sensitivity, specificity, PPV, and NPV of 100% (95% CI = 72.2, 100) even when using a wide range of prevalence estimates. However, the in-house nucleocapsid (U of T) assay only achieved a sensitivity, specificity, PPV, and NPV of 40% (95% CI = 16.8, 68.7), 100% (95% CI = 72.2, 100), 100% (95% CI = 51, 100), and 62.5% (95% CI = 38.6, 81.5) respectively suggesting that this assay would yield a high number of false negatives in high prevalence settings (Fig 1). All the in-house assays, apart from the in-house nucleocapsid (U of T) had almost perfect agreement with EUROIMMUN (Table 1) and other high performing commercial assays (S1 Table, Fig 2).

### Separation between positive and negative samples

**Fig 3:**
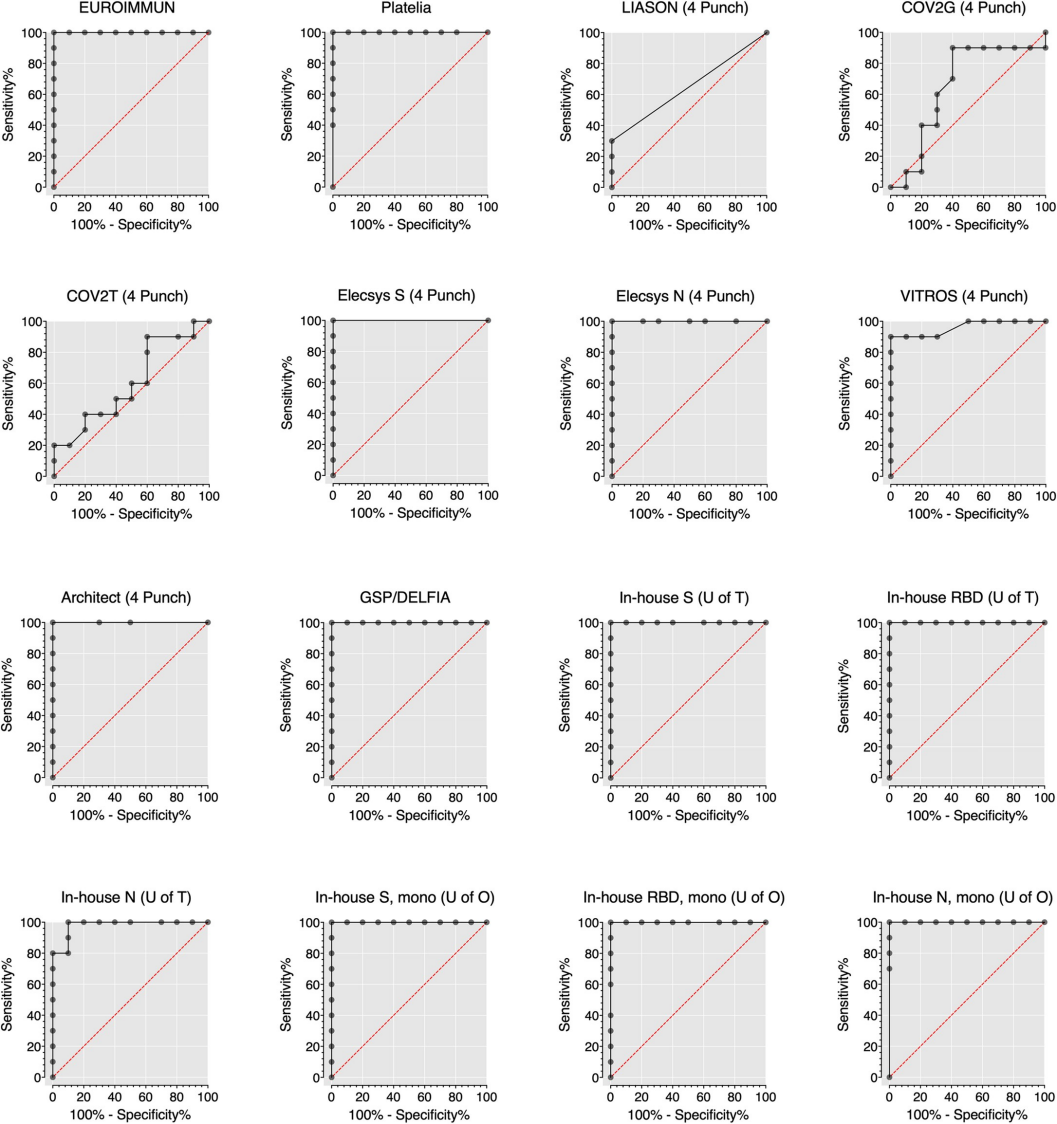
Receiver operating characteristic (ROC) curve for each commercial and in-house assay on dried blood spot specimens. ROC curves are presented for n = 10 SARS-CoV-2 antibody negative DBS specimens and n = 10 SARS-CoV-2 antibody positive DBS specimens. One 6 mm (1/4 inch) punch was used for the EUROIMMUN assay and two 6 mm (1/4 inch) punches were used for the Platelia and in-house assays. Data from four 6 mm (1/4 inch) punches is presented for all other assays. EUROIMMUN = Anti-SARS-CoV-2 ELISA assay (EUROIMMUN, Lübeck, Germany). Platelia = SARS-CoV-2 assay (Bio-Rad, Hercules, California). LIASON = SARS-CoV-2 assay (DiaSorin, Saluggia, Italy). COV2G = SARS-CoV-2 COV2G assay (Siemens, Erlangen, Germany). COV2T = SARS-CoV-2 COV2T assay (Siemens). Elecsys S = Quantitative Anti-SARS-CoV-2 assay (Elecsys spike; Roche, Basel, Switzerland). Elecsys N = Anti-SARS-CoV-2 assay (Elecsys nucleocapsid; Roche). VITROS = Anti-SARS-CoV-2 assay (Ortho Clinical Diagnostics, Raritan, New Jersey). Architect = SARS-CoV-2 assay (Abbott, Mississauga, Canada). GSP/DELFIA = Anti-SARS-CoV-2 assay (PerkinElmer, Waltham, Massachusetts). In-house S (U of T) = In-house spike assay (University of Toronto). In-house RBD (U of T) = In-house RBD assay (University of Toronto). In-house N (U of T) = In-house nucleocapsid assay (University of Toronto). In-house S, mono (U of O) = In-house monoclonal spike assay (University of Ottawa). In-house RBD, mono (U of O) = In-house monoclonal RBD assay (University of Ottawa). In-house N, mono (U of O) = In-house monoclonal nucleocapsid assay (University of Ottawa).

**Fig 4:**
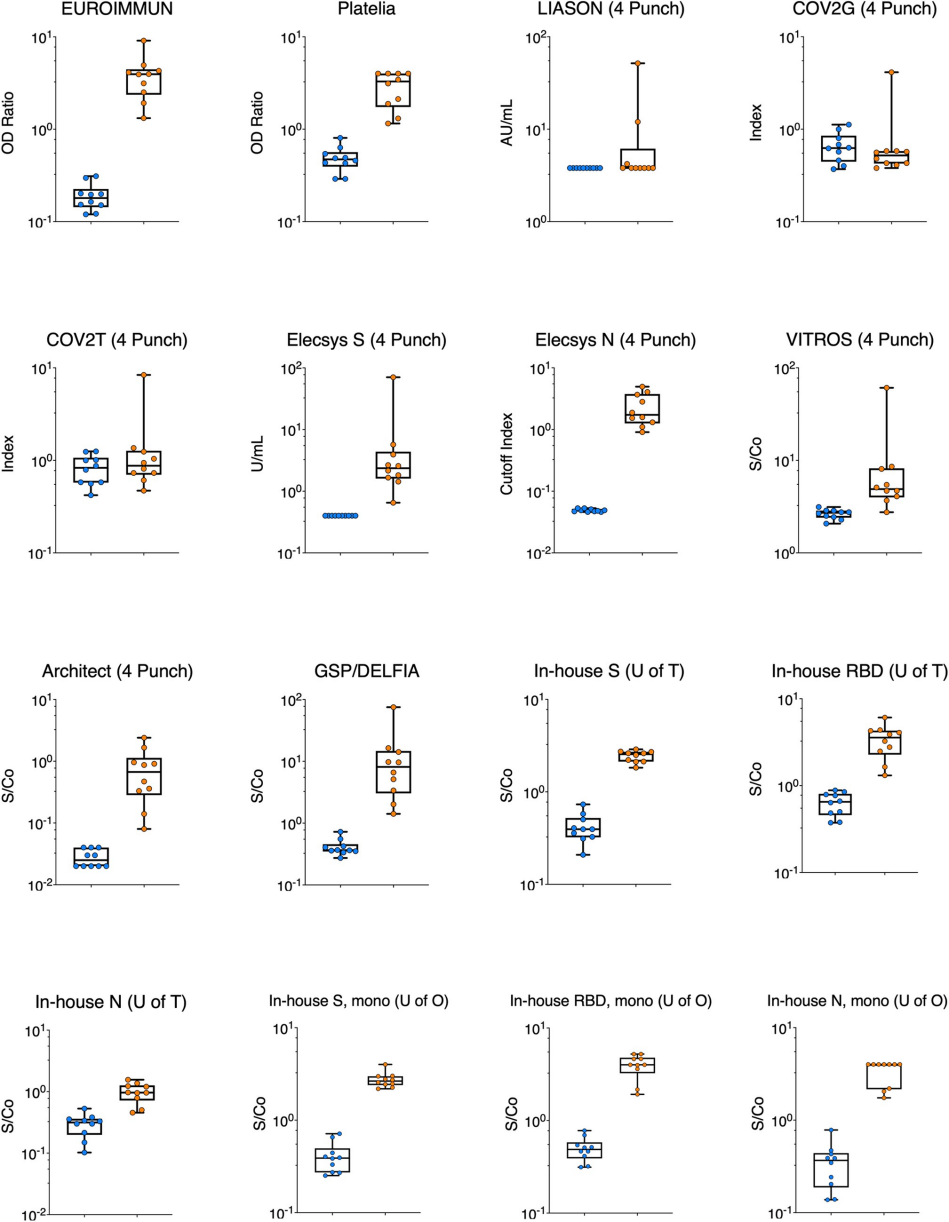
Distribution of values obtained for each commercial and in-house assay on dried blood spot (DBS) specimens. Each panel shows the optical density ratio (OD Ratio), arbitrary units per mL (AU/mL), index, units per mL (U/mL), cut-off index, or signal to cut-off ratio (S/Co) for SARS-CoV-2 antibody negative DBS specimens (n = 10) represented in blue and SARS-CoV-2 antibody positive DBS specimens (n = 10) represented in orange. All values are log_10_ transformed to aid with visualisation. One 6 mm (1/4 inch) punch was used for the EUROIMMUN assay and two 6 mm (1/4 inch) punches were used for the Platelia and in-house assays. Data from four 6 mm (1/4 inch) punches is presented for all other assays. EUROIMMUN = Anti-SARS-CoV-2 ELISA assay (EUROIMMUN, Lübeck, Germany). Platelia = SARS-CoV-2 assay (Bio-Rad, Hercules, California). LIASON = SARS-CoV-2 assay (DiaSorin, Saluggia, Italy). COV2G = SARS-CoV-2 COV2G assay (Siemens, Erlangen, Germany). COV2T = SARS-CoV-2 COV2T assay (Siemens). Elecsys S = Quantitative Anti-SARS-CoV-2 assay (Elecsys spike; Roche, Basel, Switzerland). Elecsys N = Anti-SARS-CoV-2 assay (Elecsys nucleocapsid; Roche). VITROS = Anti-SARS-CoV-2 assay (Ortho Clinical Diagnostics, Raritan, New Jersey). Architect = SARS-CoV-2 assay (Abbott, Mississauga, Canada). GSP/DELFIA = Anti-SARS-CoV-2 assay (PerkinElmer, Waltham, Massachusetts). In-house S (U of T) = In-house spike assay (University of Toronto). In-house RBD (U of T) = In-house RBD assay (University of Toronto). In-house N (U of T) = In-house nucleocapsid assay (University of Toronto). In-house S, mono (U of O) = In-house monoclonal spike assay (University of Ottawa). In-house RBD, mono (U of O) = In-house monoclonal RBD assay (University of Ottawa). In-house N, mono (U of O) = In-house monoclonal nucleocapsid assay (University of Ottawa).

Raw data values were plotted, and receiver operating characteristic (ROC) curves were computed (Figs 3 and 4, Table 2, S2 and S3 Figs) to evaluate each assay’s ability to separate the 10 known negative plasma samples and 10 plasma samples from COVID-19 patients contrived as DBS. Among the commercial assays, the EUROIMMUN, Platelia, Elecsys spike, Elecsys nucleocapsid, and Architect showed the clearest separation of positive and negative samples by DBS testing and their overall performance was rated as excellent (Table 2). On the other hand, the LIASON, COV2G, and COV2T could not clearly distinguish between positive and negative DBS specimens and showed an overall performance ranging from bad to sufficient (Table 2). The VITROS assay showed good separation of positive and negative DBS and excellent performance according to the area under the ROC curve (AUC) suggesting that increasing the signal to cut-off (S/Co) ratio from 1.0 to 3.4 could potentially increase this assay’s sensitivity and specificity to 90% (95% CI = 60, 99) and 100% (95% CI = 72.2, 100) respectively.

**Table 2:** Area under the receiver operating characteristic curve (AUC) for each commercial and in-house assay on dried blood spot specimens (DBS).

Assay	DBS punches (*n* =)	AUC^a^ (95% CI [LL, UL])	P value	Performance
**EUROIMMUN**	2	1.0 (1.0, 1.0)	<0.05	Excellent
**Platelia**	2	1.0 (1.0, 1.0)	<0.05	Excellent
**LIASON**	2	0.60 (0.35, 0.85)	0.45	Bad
**LIASON**	3	0.60 (0.35, 0.85)	0.45	Bad
**LIASON**	4	0.65 (0.40, 0.90)	0.26	Sufficient
**COV2G**	2	0.59 (0.33, 0.85)	0.50	Bad
**COV2G**	3	0.66 (0.40, 0.91)	0.24	Sufficient
**COV2G**	4	0.66 (0.39, 0.92)	0.24	Sufficient
**COV2T**	2	0.59 (0.33, 0.85)	0.50	Bad
**COV2T**	3	0.55 (0.29, 0.81)	0.71	Bad
**COV2T**	4	0.61 (0.35, 0.86)	0.43	Sufficient
**Elecsys S**	2	0.95 (0.84, 1.0)	<0.05	Excellent
**Elecsys S**	3	1.0 (1.0, 1.0)	<0.05	Excellent
**Elecsys S**	4	1.0 (1.0, 1.0)	<0.05	Excellent
**Elecsys N**	2	1.0 (1.0, 1.0)	<0.05	Excellent
**Elecsys N**	3	1.0 (1.0, 1.0)	<0.05	Excellent
**Elecsys N**	4	1.0 (1.0, 1.0)	<0.05	Excellent
**VITROS**	2	0.93 (0.78, 1.0)	<0.05	Excellent
**VITROS**	3	0.93 (0.81, 1.0)	<0.05	Excellent
**VITROS**	4	0.96 (0.87, 1.0)	<0.05	Excellent
**Architect**	2	1.0 (1.0, 1.0)	<0.05	Excellent
**Architect**	3	1.0 (1.0, 1.0)	<0.05	Excellent
**Architect**	4	1.0 (1.0, 1.0)	<0.05	Excellent
**GSP/DELFIA**	2	1.0 (1.0, 1.0)	<0.05	Excellent
**In-house S (U of T)**	2	1.0 (1.0, 1.0)	<0.05	Excellent
**In-house RBD (U of T)**	2	1.0 (1.0, 1.0)	<0.05	Excellent
**In-house N (U of T)**	2	0.98 (0.93, 1.0)	<0.05	Excellent
**In-house S, polyclonal (U of O)**	2	1.0 (1.0, 1.0)	<0.05	Excellent
**In-house RBD, polyclonal (U of O)**	2	1.0 (1.0, 1.0)	<0.05	Excellent
**In-house N, polyclonal (U of O)**	2	1.0 (1.0, 1.0)	<0.05	Excellent
**In-house S, monoclonal (U of O)**	2	1.0 (1.0, 1.0)	<0.05	Excellent
**In-house RBD, monoclonal (U of O)**	2	1.0 (1.0, 1.0)	<0.05	Excellent
**In-house N, monoclonal (U of O)**	2	1.0 (1.0, 1.0)	<0.05	Excellent

DBS punches: 6 mm (1/4 inch) dried blood spot punch; 95% CI (LL, UL): 95% confidence intervals (lower limit, upper limit); S: Spike; RBD: Receptor binding domain; N: Nucleocapsid; U of T: University of Toronto; U of O: University of Ottawa.

^a^Test performance classified according to AUC as follows: <0.5 = not useful, 0.5–0.6 = bad, 0.6–0.7 = sufficient, 0.7–0.8 = good, 0.8–0.9 = very good, and 0.9–1.0 = excellent.

The panel consisted of 10 unique SARS-CoV-2 antibody positive DBS cards and 10 unique SARS-CoV-2 negative DBS cards contrived from patient samples.

The in-house assays from the University of Ottawa and University of Toronto (spike, RBD, and nucleocapsid) showed a clear separation between positive and negative DBS (Figs 3 and 4, Table 2, S2 and S3 Figs). However, the in-house nucleocapsid (University of Toronto) assay was unable to differentiate all positive and negative DBS successfully.

## Discussion

On a small panel of DBS, this study has shown that the EUROIMMUN, Elecsys spike, and GSP/DELFIA commercial assays as well in-house assays are capable of achieving a sensitivity, specificity, PPV, and NPV of 100% (95% CI = 72.2, 100) under a broad range of prevalence scenarios following testing of DBS eluate samples collected ≥36 days post symptom onset without optimization. We interpret these findings cautiously since a recent systematic review by Lisboa Bastos *et al*. [22] assessing 40 serological studies reported that the maximum pooled sensitivities for enzyme linked immunosorbent assays measuring IgG or IgM (ELISAs) and chemiluminescent immunoassays (CLIAs) was 84.3% (95% CI = 75.6, 90.9) and 97.8% (95% CI = 46.2, 100) respectively. The accuracy of serological assays also varies according to sampling time frames and sensitivity typically exceeds 90% between 15–35 days post symptom onset [22]. We were unable to stratify our analysis according to days post symptom onset due to our sample size and narrow range of collection dates. Furthermore, our samples consisted of COVID-19 convalescent plasma donors who are likely to have significant antibody titers [23]. Nonetheless, we clearly show that certain assays like the LIASON, COV2G, and COV2T could not reliably identify positive and negative specimens by DBS testing and are unlikely to be useful in various prevalence settings.

Based on our ROC curve analysis, assays like the Platelia, Elecsys nucleocapsid, VITROS, and Architect, which did not reach 100% for all performance characteristics, could clearly distinguish between positive and negative samples therefore could also potentially achieve a sensitivity, specificity, PPV, and NPV near 100% via threshold adjustments [24–26]. However, establishing appropriate thresholds will require a study using a large number of well characterised clinical samples representing a broad range of factors associated with developing robust immune responses to SARS-CoV-2 such as sex, age, immunodeficiencies, and disease severity [27].

This validation study has several limitations that must be considered. First, our panel was small, albeit well characterized. Current efforts are underway to validate in-house and commercial multiplex assays using a larger DBS panel that covers a broader range of days between infection and sample collection. Second, we did not take into consideration other capillary blood collection methods such as capillary tubes. While capillary tubes have been shown to be a practical alternative to venepuncture for SARS-CoV-2 antibody testing [28,29], they cannot be transported as easily or safely as DBS therefore their applicability are somewhat limited for large sero-surveys within the Canadian context. Last, DBS eluates, prepared using a well-characterized buffer used specifically by the National Laboratory for HIV Reference Services (NLHRS), were sent to the participants (with the exception of the GSP/DELFIA). The assessment of different buffers or the further optimization of DBS elution conditions was beyond the scope of this study. Additional refinement of these procedures may have yielded more optimistic results on the assays that were not reported to fare well in this evaluation. However, the purpose of this evaluation was to develop a simplified procedure based on simple well-characterized practices previously developed within the NLHRS. Implementing this standardized approach allowed for an effective and timely identification of promising platforms that could be used for DBS testing for anti-SARS-CoV-2 antibodies without extensive modification. We believe that this was accomplished despite these limitations.

In conclusion, we assessed the performance of 10 commercial and 2 in-house serological assays for DBS eluate testing. Several of these assays achieved a specificity, sensitivity, PPV, and NPV adequate for sero-surveys even in low-prevalence settings. These findings suggest that the high demand for SARS-CoV-2 serology testing, mainly driven by sero-surveys within the Canadian context, could be met by the collection and testing of DBS by several different assays, thereby minimizing the risk of shortages. Furthermore, DBS collection has the potential to expand testing use and access while limiting the requirements for specimen collection on healthcare professionals already overtaxed with the COVID-19 response. While our sample size was small, this validation study, undertaken initially to determine the feasibility of a nationally representative household-based sero-survey that would be based on DBS, resulted in the selection of the in-house assays. They were chosen not only because of their test performance, but also because they are capable of distinguishing between infection- and vaccine-induced antibody responses which will be important in informing public health strategies in diverse jurisdictions across Canada. Our study represents the foundation for future validation studies on DBS specimens, that will undoubtedly play a central role in shaping Canada’s public health policy in response to COVID-19. However, larger DBS panels representing a broader range of days between symptom onset and sample collection as well as socio-demographic and clinical characteristics of COVID-19 patients will be required to substantiate our findings.

## Methods

### Ethics statement

All experiments were carried out in accordance with relevant guidelines and regulations. Written informed consent was obtained from all participants who provided blood samples from which we contrived SARS-CoV-2 antibody positive and negative DBS cards. All participants were 18 years of age or older. Ethical approval was obtained from the Health Canada and Public Health Agency of Canada Research Ethics Board (REB no. 2020-022P).

### Patient specimens

SARS-CoV-2 antibody positive and negative plasma used to contrive DBS specimens are described in Table 3. SARS-CoV-2 antibody positive plasma was collected from COVID-19 convalescent donors at Mount Sinai Hospital (Toronto, Canada). Plasma was tested for SARS-CoV-2 antibodies using the Platelia SARS-CoV-2 Total Ab (Bio-Rad, Hercules, California) or Anti-SARS-CoV-2 ELISA IgG (EUROIMMUN, Lübeck, Germany) kits. SARS-CoV-2 negative plasma was collected from healthy donors within the National Microbiology Laboratory (Winnipeg, Canada). To prepare plasma, blood was collected in EDTA Vacutainer tubes (Beckton Dickinson, Franklin Lates, NJ) and centrifuged at 1,500 RPM for 7 minutes. Plasma was tested using the Platelia SARS-CoV-2 Total Ab (Bio-Rad) or Anti-SARS-CoV-2 ELISA IgG (EUROIMMUN, Lübeck, Germany) kits to verify donors were negative for SARS-CoV-2 antibodies. All assays were performed according to the manufacturer’s instructions.

**Table 3:** SARS-CoV-2 antibody positive and negative plasma used to contrive dried blood spot specimens.

Sample ID	Description	Sample collection, days post infection	EUROIMMUN Anti-SARS-CoV-2		Bio-Rad Platelia SARS-CoV-2	
			OD ratio	Interpretation^a^	OD_M_R4 ratio	Interpretation^b^
**J12**	Healthy donor	Pre-COVID-19	0.20	Negative	0.08	Negative
**D17**	Healthy donor	Pre-COVID-19	0.12	Negative	0.11	Negative
**J11**	Healthy donor	Pre-COVID-19	0.16	Negative	0.08	Negative
**J1**	Healthy donor	Pre-COVID-19	0.15	Negative	0.08	Negative
**D142**	Healthy donor	Pre-COVID-19	0.13	Negative	0.09	Negative
**J19**	Healthy donor	Pre-COVID-19	0.49	Negative	0.08	Negative
**D9**	Healthy donor	Pre-COVID-19	0.18	Negative	0.09	Negative
**J30**	Healthy donor	Pre-COVID-19	0.17	Negative	0.10	Negative
**J15**	Healthy donor	Pre-COVID-19	0.18	Negative	0.07	Negative
**J7**	Healthy donor	Pre-COVID-19	0.41	Negative	0.10	Negative
**20–1887**	COVID-19 convalescent donor	44	2.45	Positive	≥4.00	Positive
**20–1954**	COVID-19 convalescent donor	57	2.32	Positive	3.14	Positive
**20–1955**	COVID-19 convalescent donor	42	1.36	Positive	2.12	Positive
**20–1877**	COVID-19 convalescent donor	41	4.10	Positive	≥4.00	Positive
**20–1878**	COVID-19 convalescent donor	51	4.01	Positive	≥4.00	Positive
**20–1882**	COVID-19 convalescent donor	66	2.90	Positive	≥4.00	Positive
**20–1886**	COVID-19 convalescent donor	52	4.62	Positive	≥4.00	Positive
**20–1879**	COVID-19 convalescent donor	36	11.04	Positive	≥4.00	Positive
**20–1885**	COVID-19 convalescent donor	38	5.78	Positive	≥4.00	Positive
**20–1952**	COVID-19 convalescent donor	42	4.39	Positive	≥4.00	Positive

OD: Optical density; OD_M_R4: Optical density for cut-off control R4.

^a^Results were evaluated by calculating the ratio of the OD of the plasma specimen over the OD of the assay calibrator. Results were interpreted as follows: OD ratio <0.8 reported as positive; OD ratio ≧0.8 to <1.1 reported as borderline; OD ratio ≧1.1 reported as positive.

^b^Results were evaluated by calculating the ratio of the OD of the plasma specimen over the OD of the kit cut-off control R4. Results were interpreted as follows: OD_M_R4 ratio <0.8 reported as negative; OD_M_R4 ratio o ≧0.8 to <1.0 reported as equivocal; OD_M_R4 ratio ≧1.0 reported as positive.

### Contrived dried blood spot specimens

A panel consisting of 10 unique SARS-CoV-2 antibody positive DBS cards were contrived and 10 unique SARS-CoV-2 negative DBS cards were directly spotted from EDTA whole blood to assess the performance of commercial and in-house serological tests (S4 Fig). Each testing site was blinded to the status of the DBS cards. SARS-CoV-2 antibody positive plasma samples were contrived into DBS specimens by using blood collected from healthy donors within the National Microbiology Laboratory. SARS-CoV-2 antibody negative blood was centrifuged at 1,500 RPM for 7 minutes and the plasma was removed. The remaining red blood cells were re-suspended with SARS-CoV-2 antibody positive plasma using a 1:1 ratio and 75 μL was spotted onto each circle of a Whatman 903 Protein Saver card (GE Healthcare, Boston, MA). Spotted cards were allowed to air-dry for at least 2 hours in a biosafety cabinet and then packaged in a gas impermeable bag with a desiccant pack and a humidity indicator card. Packaged cards (maximum 10 per bag) were stored at -80°C until further testing. SARS-CoV-2 antibody negative blood was spotted directly from the EDTA Vacutainer tubes onto Whatman 903 Proteinsaver cards as described above.

### Dried blood spot elution

DBS samples were punched using a 6 mm hole punch into a 96 deep well plate. One to four 6 mm (1/4 inch) punches were added to each well and eluted in DPBS buffer (pH 7.4) containing 0.5% BSA and 0.05% Tween-20 overnight at 4°C with agitation (400 RPM). For the Anti-SARS-CoV-2 ELISA IgG (EUROIMMUN) a single punch was eluted in 500 ul of Sample Buffer overnight without agitation and 100 μL of eluate was transferred to the assay plate. For Platelia SARS-CoV-2 Total Ab (Bio-Rad) assay, two punches were eluted in 130 μL of Sample Diluent overnight with gentle agitation (400 RPM), 75 μL of eluate was mixed with conjugate, and 100 μL of the eluate-conjugate mixture was transferred to the assay plate. Elution volumes were adjusted for all other assays according to manufacturer’s specifications or in-house developed protocols (Table 4). Afterwards, plates were incubated at room temperature for 30 minutes with agitation (400 RPM) and each DBS eluate was transferred to an individual 2.0 mL screw cap tube. Eluates were stored at -80°C until shipment on dry ice to each testing site.

**Table 4:** Commercial and in-house serological tests assessed for SARS-CoV-2 antibody testing on dried blood spot specimens.

Assay	Manufacturer	Site	Antibody class	Target	Elution volume (μL)^a^	Interpretation		
						Negative	Equivocal	Positive
Anti-SARS-CoV-2 ELISA	EUROIMMUN	J.C. Wilt	IgG	S1	500	OD ratio <0.8	OD ratio ≥0.8 <1.1	OD ratio ≥1.1
LIASON SARS-CoV-2	DiaSorin	Cadham	IgG	S1, S2	200	<15.0 AU/mL	N/A	≥15.0 AU/mL
SARS-CoV-2 COV2G	Siemens	U of T (DID)	IgG	S1	300	Index <1.0	N/A	Index ≥1.0
SARS-CoV-2 COV2T	Siemens	U of T (DID)	Total	S1	300	Index <1.0	N/A	Index ≥1.0
Architect SARS-CoV-2	Abbott	McGill	IgG	N	180	S/Co <1.40	N/A	S/Co ≥1.40
GSP/DELFIA Anti-SARS-CoV-2	Perkin Elmer	CHEO	IgG	S1	N/A^b^	S/Co <1.4	N/A	S/Co ≥1.4
Platelia SARS-CoV-2	Bio-Rad	J.C. Wilt	Total	N	130	OD ratio <0.8	OD ratio ≥0.8 <1.0	OD ratio ≥1.1
Elecsys Anti-SARS-CoV-2	Roche	NML	Total	S	250	<0.8 U/mL	N/A	≥0.8 U/mL
Elecsys Anti-SARS-CoV-2	Roche	NML	Total	N	250	Cutoff index <1.0	N/A	Cutoff index ≥1.0
VITROS Anti-SARS-CoV-2	Ortho Clinical Diagnostics	BCCDC	Total	S1	300	S/Co <1.0	N/A	S/Co ≥1.0
Abe KT, *et al*. 2020[30]	In-house	U of T	IgG	S	100	S/Co <1.0	N/A	S/Co ≥1.0
Abe KT, *et al*. 2020[30]	In-house	U of T	IgG	RBD	100	S/Co <1.0	N/A	S/Co ≥1.0
Abe KT, *et al*. 2020[30]	In-house	U of T	IgG	N	100	S/Co <1.0	N/A	S/Co ≥1.0
Adapted from Amanat F, *et al*. 2020[31]	In-house	U of O	IgG (polyclonal)	S	100	S/Co <1.0	N/A	S/Co ≥1.0
Adapted from Amanat F, *et al*. 2020[31]	In-house	U of O	IgG (monoclonal)	S	100	S/Co <1.0	N/A	S/Co ≥1.0
Adapted from Amanat F, *et al*. 2020[31]	In-house	U of O	IgG (polyclonal)	RBD	100	S/Co <1.0	N/A	S/Co ≥1.0
Adapted from Amanat F, *et al*. 2020[31]	In-house	U of O	IgG (monoclonal)	RBD	100	S/Co <1.0	N/A	S/Co ≥1.0
Adapted from Amanat F, *et al*. 2020[31]	In-house	U of O	IgG (polyclonal)	N	100	S/Co <1.0	N/A	S/Co ≥1.0
Adapted from Amanat F, *et al*. 2020[31]	In-house	U of O	IgG (monoclonal)	N	100	S/Co <1.0	N/A	S/Co ≥1.0

J.C. Wilt: National Microbiology Laboratory at the J.C. Wilt Infectious Diseases Research Centre, Winnipeg, Canada; U of T (DID): Division of Infectious Diseases, University of Toronto; McGill: McGill University Health Centre, McGill University; CHEO: Children’s Hospital of Eastern Ontario, Ottawa, Canada; NML: National Microbiology Laboratory at the Canadian Science Centre for Human and Animal Health, Winnipeg, Canada; BCCDC: British Columbia Centre for Disease Control, Vancouver, Canada; U of T: University of Toronto, Toronto, Canada; U of O: University of Ottawa, Ottawa, Canada; S1: Spike S1; S2: Spike S2; S: Total spike; N: Nucleocapsid; RBD: Receptor binding domain; OD: Optical density; S/Co: Signal cut-off; AU/mL: Arbitrary units/mL; N/A: Not applicable.

^a^ The elution volume was based on the minimum volume required to perform the assay, the assay’s dead volume, and an additional 30 μL of buffer for each 6 mm (1/4 inch) DBS punch to account for liquid absorbed by the filter paper.

^b^DBS cards shipped directly to testing site. The GSP/DELFIA performs automated, onboard DBS punching (1 x 3.2 mm) and elution.

### SARS-CoV-2 antibody testing

DBS eluates were tested for SARS-CoV-2 antibodies with 10 commercial assays and 2 in-house assays (Table 4) according to the manufacturer’s instructions or laboratory developed protocols respectively. No attempts were made at this point to optimize protocols for DBS specimens. The commercial tests consisted of 6 IgG based assays: Anti-SARS-CoV-2 ELISA (EUROIMMUN), LIASON SARS-CoV-2 (DiaSorin, Saluggia, Italy), SARS-CoV-2 COV2G (Siemens, Erlangen, Germany), Architect SARS-CoV-2 (Abbott, Mississauga, Canada), and GSP/DELFIA Anti-SARS-CoV-2 (PerkinElmer, Waltham, Massachusetts); and 5 total antibody assays: Platelia SARS-CoV-2 (Bio-Rad), SARS-CoV-2 COV2T (Siemens), Elecsys quantitative Anti-SARS-CoV-2 (Elecsys spike; Roche, Basel, Switzerland), Elecsys Anti-SARS-CoV-2 (Elecsys nucleocapsid; Roche), and VITROS Anti-SARS-CoV-2 (Ortho Clinical Diagnostics, Raritan, New Jersey). EUROIMMUN was included since it is the only assay currently approved by Health Canada for use with serum which also has a manufacturer developed protocol for use with DBS (CE marked). Both in-house tests, described in greater detail elsewhere [30] and Table 2, consist of IgG assays targeting SARS-CoV-2 spike, receptor binding domain (RBD), and nucleocapsid proteins. Each testing site was responsible for interpreting and reporting their own data.

### Statistical analysis

Statistical analysis was conducted using Prism version 9.0.0 (GraphPad Software, San Diego, CA). Test performance on DBS expressed in terms of sensitivity, specificity, positive predictive values (PPV), and negative predictive values (NPV), was computed in comparison with the serum-based Anti-SARS-CoV-2 ELISA (EUROIMMUN) assay results as the reference because it is the only assay currently approved by Health Canada for use with serum which also has a manufacturer developed protocol for use with DBS (CE marked). PPV (sensitivity x prevalence/sensitivity x prevalence + [1 –specificity] x [1 –prevalence]) and NPV (specificity x [1 –prevalence]/specificity x [1 –prevalence] + [1 –sensitivity) x prevalence) was also re-calculated using a prevalence of 1.0%, 2.5%, 5.0%, 10.0%, 20.0%, and 40.0%. Equivocal results were considered positive.

Agreement with the reference test was quantified using Cohen’s kappa coefficient (https://www.graphpad.com/quickcalcs/kappa1/?K=2) and interpreted as follows: <0 = no agreement, 0–0.20 = slight agreement, 0.21–0.40 = fair agreement, 0.41–0.60 = moderate agreement, 0.61–0.80 = substantial agreement, and 0.81–1.00 = almost perfect agreement [32]. Confidence intervals were computed using the hybrid Wilson/Brown method [33].

Receiver operating characteristics (ROC) curves were also computed using the Wilson/Brown method. A P-value ≤0.05 was considered statistically significant. Test performance classified according to the area under the ROC curve (AUC) was interpreted as follows: <0.5 = not useful, 0.5–0.6 = bad, 0.6–0.7 = sufficient, 0.7–0.8 = good, 0.8–0.9 = very good, and 0.9–1.0 = excellent [34,35].

Overall positive and negative percent concordance and Cohen’s kappa coefficient were used to quantify inter-assay agreement between the 10 commercial assays and 2 in-house assays. Confidence intervals were computed using the hybrid Wilson/Brown method. Cohen’s kappa coefficient was interpreted as described above.

## Supporting information

S1 Fig Positive predictive values (PPV) and negative predictive values (NPV) by prevalence for each commercial and in-house assay on dried blood spot specimens. ROC curves are presented for n = 10 SARS-CoV-2 antibody negative DBS specimens and n = 10 SARS-CoV-2 antibody positive DBS specimens. One 6 mm (1/4 inch) punch was used for the EUROIMMUN assay and two 6 mm (1/4 inch) punches were used for the Platelia and in-house assays. EUROIMMUN = Anti-SARS-CoV-2 ELISA assay (EUROIMMUN, Lübeck, Germany). Platelia = SARS-CoV-2 assay (Bio-Rad, Hercules, California). LIASON = SARS-CoV-2 assay (DiaSorin, Saluggia, Italy). COV2G = SARS-CoV-2 COV2G assay (Siemens, Erlangen, Germany). COV2T = SARS-CoV-2 COV2T assay (Siemens). Elecsys S = Quantitative Anti-SARS-CoV-2 assay (Elecsys spike; Roche, Basel, Switzerland). Elecsys N = Anti-SARS-CoV-2 assay (Elecsys nucleocapsid; Roche). VITROS = Anti-SARS-CoV-2 assay (Ortho Clinical Diagnostics, Raritan, New Jersey). Architect = SARS-CoV-2 assay (Abbott, Mississauga, Canada). GSP/DELFIA = Anti-SARS-CoV-2 assay (PerkinElmer, Waltham, Massachusetts). In-house S (U of T) = In-house spike assay (University of Toronto). In-house RBD (U of T) = In-house RBD assay (University of Toronto). In-house N (U of T) = In-house nucleocapsid assay (University of Toronto). In-house S, mono (U of O) = In- house monoclonal spike assay (University of Ottawa). In-house RBD, mono (U of O) = In-house monoclonal RBD assay (University of Ottawa). In-house N, mono (U of O) = In-house monoclonal nucleocapsid assay (University of Ottawa). The VITROS assay could not achieve a specificity greater than 0% therefore, only PPV is shown.
(TIF)

S2 Fig Receiver operating characteristic curve for each commercial and in-house assay on dried blood spot specimens. ROC curves are presented for n = 10 SARS-CoV-2 antibody negative DBS specimens and n = 10 SARS-CoV-2 antibody positive DBS specimens. One 6 mm (1/4 inch) punch was used for the EUROIMMUN assay and two 6 mm (1/4 inch) punches were used for the Platelia and in-house assays. EUROIMMUN = Anti-SARS-CoV-2 ELISA assay (EUROIMMUN, Lübeck, Germany). Platelia = SARS-CoV-2 assay (Bio-Rad, Hercules, California). LIASON = SARS-CoV-2 assay (DiaSorin, Saluggia, Italy). COV2G = SARS-CoV-2 COV2G assay (Siemens, Erlangen, Germany). COV2T = SARS-CoV-2 COV2T assay (Siemens). Elecsys S = Quantitative Anti-SARS-CoV-2 assay (Elecsys spike; Roche, Basel, Switzerland). Elecsys N = Anti-SARS-CoV-2 assay (Elecsys nucleocapsid; Roche). VITROS = Anti-SARS-CoV-2 assay (Ortho Clinical Diagnostics, Raritan, New Jersey). Architect = SARS-CoV-2 assay (Abbott, Mississauga, Canada). GSP/DELFIA = Anti-SARS-CoV-2 assay (PerkinElmer, Waltham, Massachusetts). In-house S (U of T) = In-house spike assay (University of Toronto). In-house RBD (U of T) = In-house RBD assay (University of Toronto). In-house N (U of T) = In-house nucleocapsid assay (University of Toronto). In-house S, mono (U of O) = In- house monoclonal spike assay (University of Ottawa). In-house RBD, mono (U of O) = In-house monoclonal RBD assay (University of Ottawa). In-house N, mono (U of O) = In-house monoclonal nucleocapsid assay (University of Ottawa).
(TIF)

S3 Fig Distribution of values obtained for each commercial and in-house assay on dried blood spot (DBS) specimens. Distribution of values obtained for each commercial and in-house assay on dried blood spot (DBS) specimens. Each panel shows the optical density ratio (OD Ratio), arbitrary units per mL (AU/mL), index, units per mL (U/mL), cut-off index, or signal to cut-off ratio (S/Co) for SARS-CoV-2 antibody negative DBS specimens (n = 10) represented in blue and SARS-CoV-2 antibody positive DBS specimens (n = 10) represented in orange. All values are log_10_ transformed to aid with visualisation. One 6 mm (1/4 inch) punch was used for the EUROIMMUN assay, and two 6 mm (1/4 inch) punches were used for the Platelia and in-house assays. EUROIMMUN = Anti-SARS-CoV-2 ELISA assay (EUROIMMUN, Lübeck, Germany). Platelia = SARS-CoV-2 assay (Bio-Rad, Hercules, California). LIASON = SARS-CoV-2 assay (DiaSorin, Saluggia, Italy). COV2G = SARS-CoV-2 COV2G assay (Siemens, Erlangen, Germany). COV2T = SARS-CoV-2 COV2T assay (Siemens). Elecsys S = Quantitative Anti-SARS-CoV-2 assay (Elecsys spike; Roche, Basel, Switzerland). Elecsys N = Anti-SARS-CoV-2 assay (Elecsys nucleocapsid; Roche). VITROS = Anti-SARS-CoV-2 assay (Ortho Clinical Diagnostics, Raritan, New Jersey). Architect = SARS-CoV-2 assay (Abbott, Mississauga, Canada). GSP/DELFIA = Anti-SARS-CoV-2 assay (PerkinElmer, Waltham, Massachusetts). In-house S (U of T) = In-house spike assay (University of Toronto). In-house RBD (U of T) = In-house RBD assay (University of Toronto). In-house N (U of T) = In-house nucleocapsid assay (University of Toronto). In-house S, mono (U of O) = In-house monoclonal spike assay (University of Ottawa). In-house RBD, mono (U of O) = In-house monoclonal RBD assay (University of Ottawa). In-house N, mono (U of O) = In-house monoclonal nucleocapsid assay (University of Ottawa). In-house S, poly (U of O) = In-house polyclonal spike assay (University of Ottawa). In-house RBD, poly (U of O) = In-house polyclonal RBD assay (University of Ottawa). In-house N, poly (U of O) = In-house polyclonal nucleocapsid assay (University of Ottawa).
(TIF)

S4 Fig Procedure for contriving dried blood spot specimens. (TIF)

S1 Table Inter-assay concordance and Cohen’s kappa coefficient for SARS-CoV-2 antibody testing on dried blood spot specimens. (DOCX)

S2 Table Raw data generated during this study for each commercial and in-house assay on dried blood spot specimens. (DOCX)
