# Supplementary figures and images for: Dried blood spot specimens for SARS-CoV-2 antibody testing: A multi-site, multi-assay comparison
Source: PLoS One. 2021 Dec 7;16(12):e0261003. doi: 10.1371/journal.pone.0261003 (PMC8651133; doi:10.1371/journal.pone.0261003)

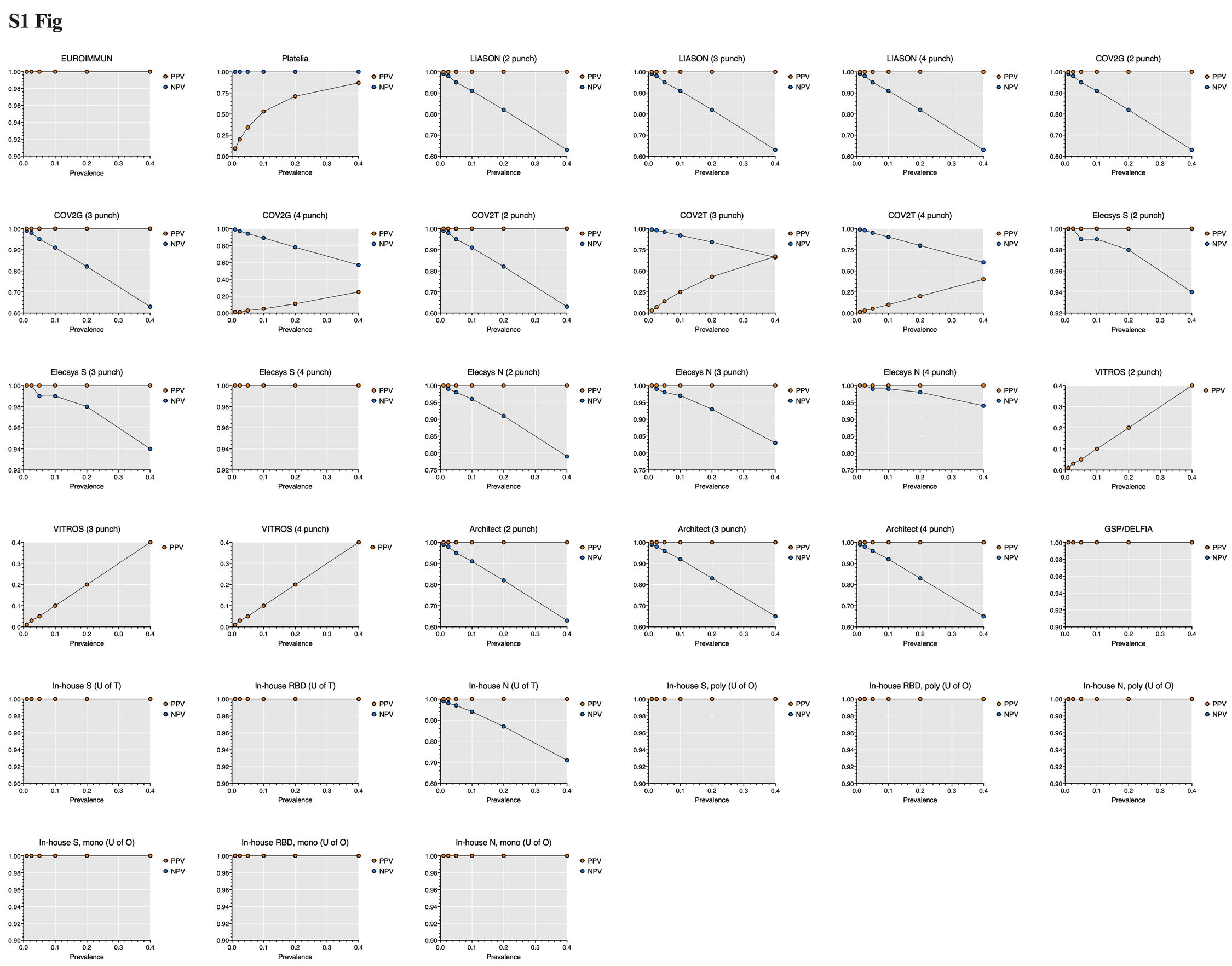

Supplement: S1 Fig — ROC curves are presented for n = 10 SARS-CoV-2 antibody negative DBS specimens and n = 10 SARS-CoV-2 antibody positive DBS specimens. One 6 mm (1/4 inch) punch was used for the EUROIMMUN assay and two 6 mm (1/4 inch) punches were used for the Platelia and in-house assays. EUROIMMUN = Anti-SARS-CoV-2 ELISA assay (EUROIMMUN, Lübeck, Germany). Platelia = SARS-CoV-2 assay (Bio-Rad, Hercules, California). LIASON = SARS-CoV-2 assay (DiaSorin, Saluggia, Italy). COV2G = SARS-CoV-2 COV2G assay (Siemens, Erlangen, Germany). COV2T = SARS-CoV-2 COV2T assay (Siemens). Elecsys S = Quantitative Anti-SARS-CoV-2 assay (Elecsys spike; Roche, Basel, Switzerland). Elecsys N = Anti-SARS-CoV-2 assay (Elecsys nucleocapsid; Roche). VITROS = Anti-SARS-CoV-2 assay (Ortho Clinical Diagnostics, Raritan, New Jersey). Architect = SARS-CoV-2 assay (Abbott, Mississauga, Canada). GSP/DELFIA = Anti-SARS-CoV-2 assay (PerkinElmer, Waltham, Massachusetts). In-house S (U of T) = In-house spike assay (University of Toronto). In-house RBD (U of T) = In-house RBD assay (University of Toronto). In-house N (U of T) = In-house nucleocapsid assay (University of Toronto). In-house S, mono (U of O) = In- house monoclonal spike assay (University of Ottawa). In-house RBD, mono (U of O) = In-house monoclonal RBD assay (University of Ottawa). In-house N, mono (U of O) = In-house monoclonal nucleocapsid assay (University of Ottawa). The VITROS assay could not achieve a specificity greater than 0% therefore, only PPV is shown. (TIF) [file pone.0261003.s001.tif]

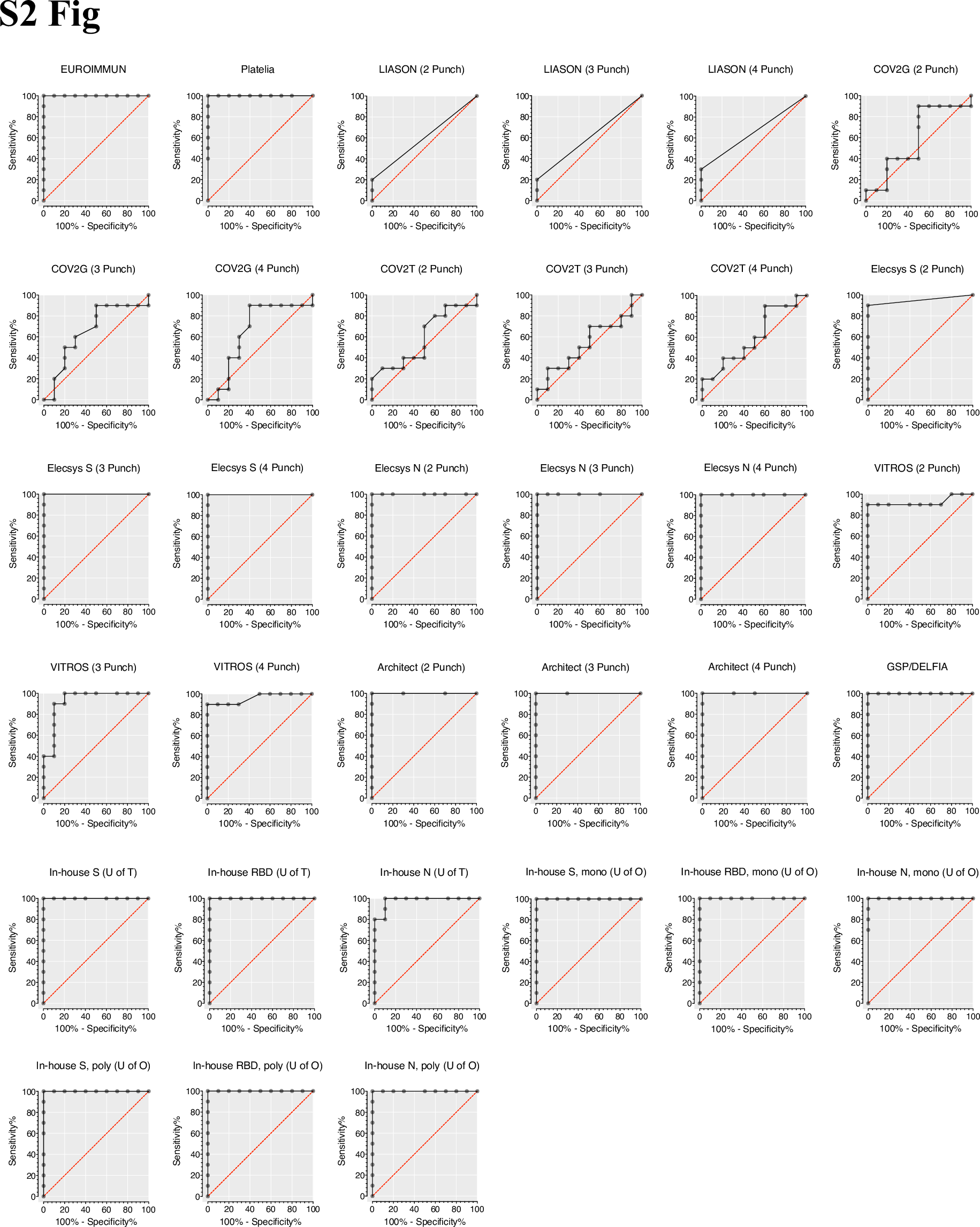

Supplement: S2 Fig — ROC curves are presented for n = 10 SARS-CoV-2 antibody negative DBS specimens and n = 10 SARS-CoV-2 antibody positive DBS specimens. One 6 mm (1/4 inch) punch was used for the EUROIMMUN assay and two 6 mm (1/4 inch) punches were used for the Platelia and in-house assays. EUROIMMUN = Anti-SARS-CoV-2 ELISA assay (EUROIMMUN, Lübeck, Germany). Platelia = SARS-CoV-2 assay (Bio-Rad, Hercules, California). LIASON = SARS-CoV-2 assay (DiaSorin, Saluggia, Italy). COV2G = SARS-CoV-2 COV2G assay (Siemens, Erlangen, Germany). COV2T = SARS-CoV-2 COV2T assay (Siemens). Elecsys S = Quantitative Anti-SARS-CoV-2 assay (Elecsys spike; Roche, Basel, Switzerland). Elecsys N = Anti-SARS-CoV-2 assay (Elecsys nucleocapsid; Roche). VITROS = Anti-SARS-CoV-2 assay (Ortho Clinical Diagnostics, Raritan, New Jersey). Architect = SARS-CoV-2 assay (Abbott, Mississauga, Canada). GSP/DELFIA = Anti-SARS-CoV-2 assay (PerkinElmer, Waltham, Massachusetts). In-house S (U of T) = In-house spike assay (University of Toronto). In-house RBD (U of T) = In-house RBD assay (University of Toronto). In-house N (U of T) = In-house nucleocapsid assay (University of Toronto). In-house S, mono (U of O) = In- house monoclonal spike assay (University of Ottawa). In-house RBD, mono (U of O) = In-house monoclonal RBD assay (University of Ottawa). In-house N, mono (U of O) = In-house monoclonal nucleocapsid assay (University of Ottawa). (TIF) [file pone.0261003.s002.tif]

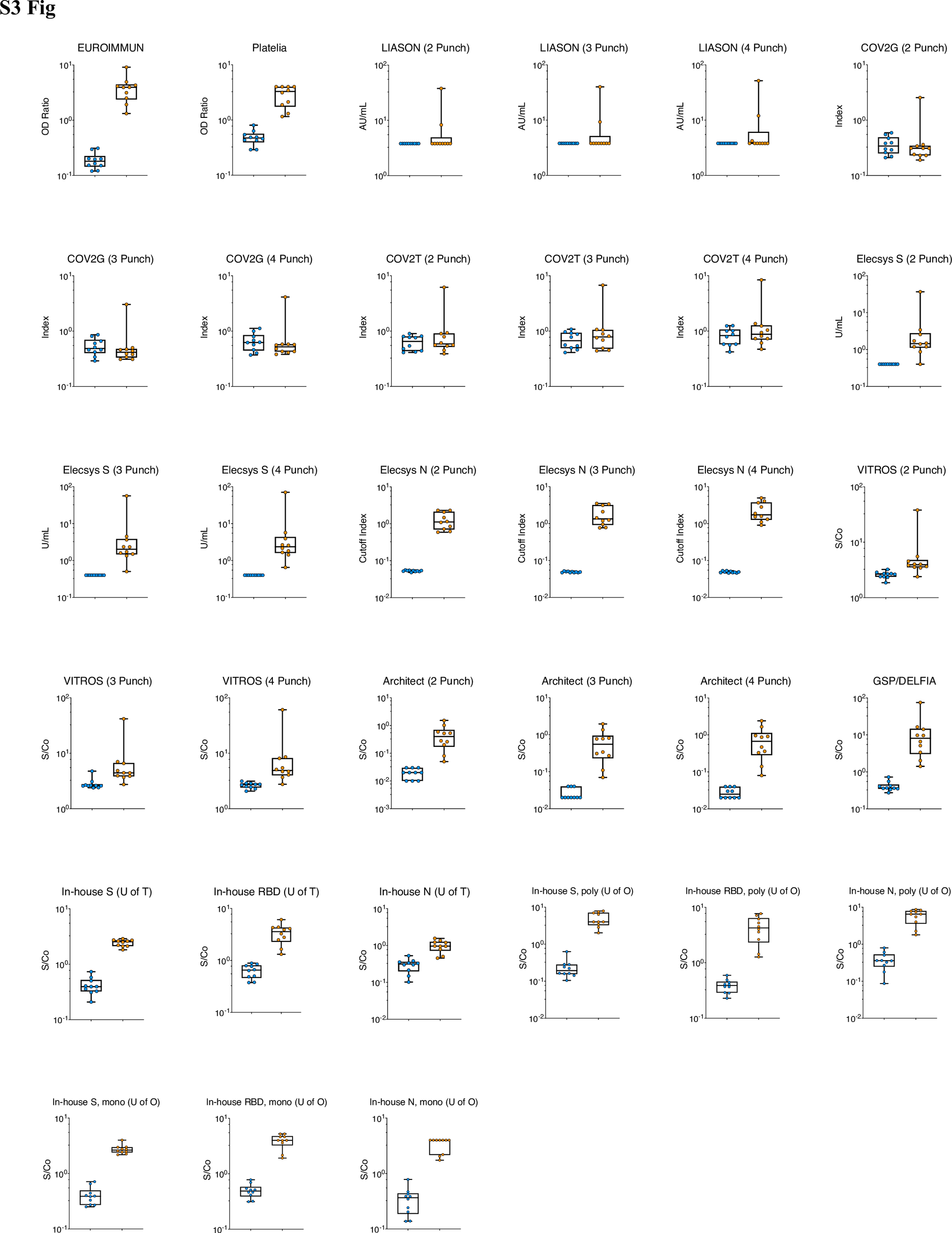

Supplement: S3 Fig — Distribution of values obtained for each commercial and in-house assay on dried blood spot (DBS) specimens. Each panel shows the optical density ratio (OD Ratio), arbitrary units per mL (AU/mL), index, units per mL (U/mL), cut-off index, or signal to cut-off ratio (S/Co) for SARS-CoV-2 antibody negative DBS specimens (n = 10) represented in blue and SARS-CoV-2 antibody positive DBS specimens (n = 10) represented in orange. All values are log10 transformed to aid with visualisation. One 6 mm (1/4 inch) punch was used for the EUROIMMUN assay, and two 6 mm (1/4 inch) punches were used for the Platelia and in-house assays. EUROIMMUN = Anti-SARS-CoV-2 ELISA assay (EUROIMMUN, Lübeck, Germany). Platelia = SARS-CoV-2 assay (Bio-Rad, Hercules, California). LIASON = SARS-CoV-2 assay (DiaSorin, Saluggia, Italy). COV2G = SARS-CoV-2 COV2G assay (Siemens, Erlangen, Germany). COV2T = SARS-CoV-2 COV2T assay (Siemens). Elecsys S = Quantitative Anti-SARS-CoV-2 assay (Elecsys spike; Roche, Basel, Switzerland). Elecsys N = Anti-SARS-CoV-2 assay (Elecsys nucleocapsid; Roche). VITROS = Anti-SARS-CoV-2 assay (Ortho Clinical Diagnostics, Raritan, New Jersey). Architect = SARS-CoV-2 assay (Abbott, Mississauga, Canada). GSP/DELFIA = Anti-SARS-CoV-2 assay (PerkinElmer, Waltham, Massachusetts). In-house S (U of T) = In-house spike assay (University of Toronto). In-house RBD (U of T) = In-house RBD assay (University of Toronto). In-house N (U of T) = In-house nucleocapsid assay (University of Toronto). In-house S, mono (U of O) = In-house monoclonal spike assay (University of Ottawa). In-house RBD, mono (U of O) = In-house monoclonal RBD assay (University of Ottawa). In-house N, mono (U of O) = In-house monoclonal nucleocapsid assay (University of Ottawa). In-house S, poly (U of O) = In-house polyclonal spike assay (University of Ottawa). In-house RBD, poly (U of O) = In-house polyclonal RBD assay (University of Ottawa). In-house N, poly (U of O) = In-house polyclonal nucleocapsid assay (Univer [file pone.0261003.s003.tif]

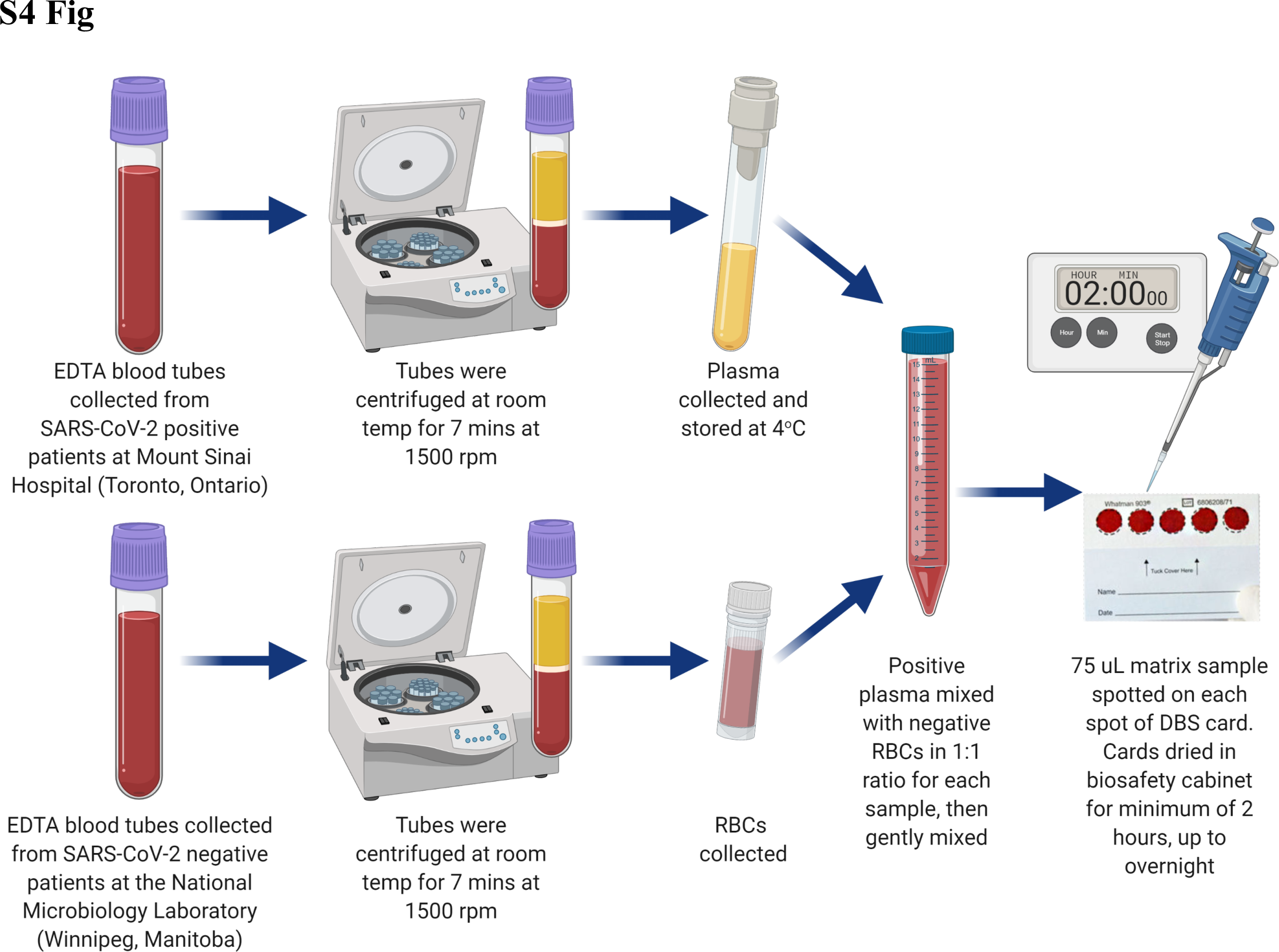

Supplement: S4 Fig — (TIF) [file pone.0261003.s004.tif]
