# Supplementary material for: Dried blood spot specimens for SARS-CoV-2 antibody testing: A multi-site, multi-assay comparison
Source: PLoS One. 2021 Dec 7;16(12):e0261003. doi: 10.1371/journal.pone.0261003 (PMC8651133; doi:10.1371/journal.pone.0261003)
Supplement: S1 Table — (DOCX) [file pone.0261003.s005.docx]

**S1 Table**

|  | **EUROIMMUN** | **Platelia** | **LIASON**  **(*n*=2)** | **LIASON**  **(*n*=3)** | **LIASON**  **(*n*=4)** | **COV2G**  **(*n*=2)** | **COV2G**  **(*n*=3)** | **COV2G**  **(*n*=4)** | **COV2T**  **(*n*=2)** | **COV2T**  **(*n*=3)** | **COV2T**  **(*n*=4)** | **Elecsys S**  **(*n*=2)** | **Elecsys S**  **(*n*=3)** | **Elecsys S**  **(*n*=4)** | **Elecsys N**  **(*n*=2)** | **Elecsys N**  **(*n*=3)** | **Elecsys N**  **(*n*=4)** | **VITROS**  **(*n*=2)** | **VITROS**  **(*n*=3)** | **VITROS**  **(*n*=4)** | **Architect**  **(*n*=2)** | **Architect**  **(*n*=3)** | **Architect**  **(*n*=4)** | **GSP/DELFIA** | **In-house S**  **(U of T)** | **In-house RBD**  **(U of T)** | **In-house N**  **(U of T)** | **In-house S,**  **poly (U of O)** | **In-house RBD,**  **poly (U of O)** | **In-house N,**  **poly (U of O)** | **In-house S,**  **mono (U of O)** | **In-house RBD,**  **mono (U of O)** | **In-house N,**  **mono (U of O)** |
| --- | --- | --- | --- | --- | --- | --- | --- | --- | --- | --- | --- | --- | --- | --- | --- | --- | --- | --- | --- | --- | --- | --- | --- | --- | --- | --- | --- | --- | --- | --- | --- | --- | --- |
| **EUROIMMUN** |  |  |  |  |  |  |  |  |  |  |  |  |  |  |  |  |  |  |  |  |  |  |  |  |  |  |  |  |  |  |  |  |  |
| Concordance (%) |  |  |  |  |  |  |  |  |  |  |  |  |  |  |  |  |  |  |  |  |  |  |  |  |  |  |  |  |  |  |  |  |  |
| Kappa (95% CI [LL, UL]) |  |  |  |  |  |  |  |  |  |  |  |  |  |  |  |  |  |  |  |  |  |  |  |  |  |  |  |  |  |  |  |  |  |
| **Platelia** |  |  |  |  |  |  |  |  |  |  |  |  |  |  |  |  |  |  |  |  |  |  |  |  |  |  |  |  |  |  |  |  |  |
| Concordance (%) | 95.0 |  |  |  |  |  |  |  |  |  |  |  |  |  |  |  |  |  |  |  |  |  |  |  |  |  |  |  |  |  |  |  |  |
| Kappa (95% CI [LL, UL]) | 0.90 (0.71, 1.00) |  |  |  |  |  |  |  |  |  |  |  |  |  |  |  |  |  |  |  |  |  |  |  |  |  |  |  |  |  |  |  |  |
| **LIASON (n=2)** |  |  |  |  |  |  |  |  |  |  |  |  |  |  |  |  |  |  |  |  |  |  |  |  |  |  |  |  |  |  |  |  |  |
| Concordance (%) | 55.0 | 52.6 |  |  |  |  |  |  |  |  |  |  |  |  |  |  |  |  |  |  |  |  |  |  |  |  |  |  |  |  |  |  |  |
| Kappa (95% CI [LL, UL]) | 0.10 (-0.09, 0.29) | 0.10 (-0.09, 0.28) |  |  |  |  |  |  |  |  |  |  |  |  |  |  |  |  |  |  |  |  |  |  |  |  |  |  |  |  |  |  |  |
| **LIASON (n=3)** |  |  |  |  |  |  |  |  |  |  |  |  |  |  |  |  |  |  |  |  |  |  |  |  |  |  |  |  |  |  |  |  |  |
| Concordance (%) | 55.0 | 52.6 | 100.0 |  |  |  |  |  |  |  |  |  |  |  |  |  |  |  |  |  |  |  |  |  |  |  |  |  |  |  |  |  |  |
| Kappa (95% CI [LL, UL]) | 0.10 (-0.09, 0.29) | 0.10 (-0.09, 0.28) | 1.00 (1.00, 1.00) |  |  |  |  |  |  |  |  |  |  |  |  |  |  |  |  |  |  |  |  |  |  |  |  |  |  |  |  |  |  |
| **LIASON (n=4)** |  |  |  |  |  |  |  |  |  |  |  |  |  |  |  |  |  |  |  |  |  |  |  |  |  |  |  |  |  |  |  |  |  |
| Concordance (%) | 55.0 | 52.6 | 100.0 | 100.0 |  |  |  |  |  |  |  |  |  |  |  |  |  |  |  |  |  |  |  |  |  |  |  |  |  |  |  |  |  |
| Kappa (95% CI [LL, UL]) | 0.10 (-0.09, 0.29) | 0.10 (-0.09, 0.28) | 1.00 (1.00, 1.00) | 1.00 (1.00, 1.00) |  |  |  |  |  |  |  |  |  |  |  |  |  |  |  |  |  |  |  |  |  |  |  |  |  |  |  |  |  |
| **COV2G (n=2)** |  |  |  |  |  |  |  |  |  |  |  |  |  |  |  |  |  |  |  |  |  |  |  |  |  |  |  |  |  |  |  |  |  |
| Concordance (%) | 55.0 | 52.6 | 100.0 | 100.0 | 100.0 |  |  |  |  |  |  |  |  |  |  |  |  |  |  |  |  |  |  |  |  |  |  |  |  |  |  |  |  |
| Kappa (95% CI [LL, UL]) | 0.10 (-0.09, 0.29) | 0.10 (-0.09, 0.28) | 1.00 (1.00, 1.00) | 1.00 (1.00, 1.00) | 1.00 (1.00, 1.00) |  |  |  |  |  |  |  |  |  |  |  |  |  |  |  |  |  |  |  |  |  |  |  |  |  |  |  |  |
| **COV2G (n=3)** |  |  |  |  |  |  |  |  |  |  |  |  |  |  |  |  |  |  |  |  |  |  |  |  |  |  |  |  |  |  |  |  |  |
| Concordance (%) | 55.0 | 52.6 | 100.0 | 100.0 | 100.0 | 100.0 |  |  |  |  |  |  |  |  |  |  |  |  |  |  |  |  |  |  |  |  |  |  |  |  |  |  |  |
| Kappa (95% CI [LL, UL]) | 0.10 (-0.09, 0.29) | 0.10 (-0.09, 0.28) | 1.00 (1.00, 1.00) | 1.00 (1.00, 1.00) | 1.00 (1.00, 1.00) | 1.00 (1.00, 1.00) |  |  |  |  |  |  |  |  |  |  |  |  |  |  |  |  |  |  |  |  |  |  |  |  |  |  |  |
| **COV2G (n=4)** |  |  |  |  |  |  |  |  |  |  |  |  |  |  |  |  |  |  |  |  |  |  |  |  |  |  |  |  |  |  |  |  |  |
| Concordance (%) | 45.0 | 42.1 | 90.0 | 90.0 | 90.0 | 90.0 | 90.0 |  |  |  |  |  |  |  |  |  |  |  |  |  |  |  |  |  |  |  |  |  |  |  |  |  |  |
| Kappa (95% CI [LL, UL]) | -0.10 (-0.41, 0.21) | -0.12 (-0.44, 0.20) | 0.46 (-0.14, 1.00) | 0.46 (-0.14, 1.00) | 0.46 (-0.14, 1.00) | 0.46 (-0.14, 1.00) | 0.46 (-0.14, 1.00) |  |  |  |  |  |  |  |  |  |  |  |  |  |  |  |  |  |  |  |  |  |  |  |  |  |  |
| **COV2T (n=2)** |  |  |  |  |  |  |  |  |  |  |  |  |  |  |  |  |  |  |  |  |  |  |  |  |  |  |  |  |  |  |  |  |  |
| Concordance (%) | 55.0 | 52.6 | 100.0 | 100.0 | 100.0 | 100.0 | 100.0 | 95.0 |  |  |  |  |  |  |  |  |  |  |  |  |  |  |  |  |  |  |  |  |  |  |  |  |  |
| Kappa (95% CI [LL, UL]) | 0.10 (-0.09, 0.29) | 0.10 (-0.09, 0.28) | 1.00 (1.00, 1.00) | 1.00 (1.00, 1.00) | 1.00 (1.00, 1.00) | 1.00 (1.00, 1.00) | 1.00 (1.00, 1.00) | 0.64 (0.01, 1.00) |  |  |  |  |  |  |  |  |  |  |  |  |  |  |  |  |  |  |  |  |  |  |  |  |  |
| **COV2T (n=3)** |  |  |  |  |  |  |  |  |  |  |  |  |  |  |  |  |  |  |  |  |  |  |  |  |  |  |  |  |  |  |  |  |  |
| Concordance (%) | 60.0 | 57.9 | 85.0 | 85.0 | 85.0 | 85.0 | 85.0 | 90.0 | 85.0 |  |  |  |  |  |  |  |  |  |  |  |  |  |  |  |  |  |  |  |  |  |  |  |  |
| Kappa (95% CI [LL, UL]) | 0.20 (-0.14, 0.54) | 0.18 (-0.16, 0.53) | 0.35 (-0.17, 0.86) | 0.35 (-0.17, 0.86) | 0.35 (-0.17, 0.86) | 0.35 (-0.17, 0.86) | 0.35 (-0.17, 0.86) | 0.62 (0.15, 1.00) | 0.35 (-0.17, 0.86) |  |  |  |  |  |  |  |  |  |  |  |  |  |  |  |  |  |  |  |  |  |  |  |  |
| **COV2T (n=4)** |  |  |  |  |  |  |  |  |  |  |  |  |  |  |  |  |  |  |  |  |  |  |  |  |  |  |  |  |  |  |  |  |  |
| Concordance (%) | 50.0 | 52.6 | 65.0 | 65.0 | 65.0 | 65.0 | 65.0 | 60.0 | 65.0 | 60.0 |  |  |  |  |  |  |  |  |  |  |  |  |  |  |  |  |  |  |  |  |  |  |  |
| Kappa (95% CI [LL, UL]) | 0 (-0.43, 0.43) | 0.07 (-0.36, 0.49) | 0.15 (-0.12, 0.41) | 0.15 (-0.12, 0.41) | 0.15 (-0.12, 0.41) | 0.15 (-0.12, 0.41) | 0.15 (-0.12, 0.41) | 0.05 (-0.27, 0.36) | 0.15 (-0.12, 0.41) | 0.09 (-0.31, 0.49) |  |  |  |  |  |  |  |  |  |  |  |  |  |  |  |  |  |  |  |  |  |  |  |
| **Elecsys S (n=2)** |  |  |  |  |  |  |  |  |  |  |  |  |  |  |  |  |  |  |  |  |  |  |  |  |  |  |  |  |  |  |  |  |  |
| Concordance (%) | 95.0 | 94.7 | 60.0 | 60.0 | 60.0 | 60.0 | 60.0 | 55.0 | 60.0 | 65.0 | 55.0 |  |  |  |  |  |  |  |  |  |  |  |  |  |  |  |  |  |  |  |  |  |  |
| Kappa (95% CI [LL, UL]) | 0.90 (0.71, 1.00) | 0.10 (0.70, 1.00) | 0.12 (-0.10, 0.35) | 0.12 (-0.10, 0.35) | 0.12 (-0.10, 0.35) | 0.12 (-0.10, 0.35) | 0.12 (-0.10, 0.35) | 0.02 (-0.27, 0.31) | 0.12 (-0.10, 0.35) | 0.26 (-0.12, 0.63) | 0.08 (-0.35, 0.52) |  |  |  |  |  |  |  |  |  |  |  |  |  |  |  |  |  |  |  |  |  |  |
| **Elecsys S (n=3)** |  |  |  |  |  |  |  |  |  |  |  |  |  |  |  |  |  |  |  |  |  |  |  |  |  |  |  |  |  |  |  |  |  |
| Concordance (%) | 95.0 | 94.7 | 60.0 | 60.0 | 60.0 | 60.0 | 60.0 | 55.0 | 60.0 | 65.0 | 55.0 | 100.0 |  |  |  |  |  |  |  |  |  |  |  |  |  |  |  |  |  |  |  |  |  |
| Kappa (95% CI [LL, UL]) | 0.90 (0.71, 1.00) | 0.10 (0.70, 1.00) | 0.12 (-0.10, 0.35) | 0.12 (-0.10, 0.35) | 0.12 (-0.10, 0.35) | 0.12 (-0.10, 0.35) | 0.12 (-0.10, 0.35) | 0.02 (-0.27, 0.31) | 0.12 (-0.10, 0.35) | 0.26 (-0.12, 0.63) | 0.08 (-0.35, 0.52) | 1.00 (1.00, 1.00) |  |  |  |  |  |  |  |  |  |  |  |  |  |  |  |  |  |  |  |  |  |
| **Elecsys S (n=4)** |  |  |  |  |  |  |  |  |  |  |  |  |  |  |  |  |  |  |  |  |  |  |  |  |  |  |  |  |  |  |  |  |  |
| Concordance (%) | 100.0 | 100.0 | 55.0 | 55.0 | 55.0 | 55.0 | 55.0 | 50.0 | 55.0 | 60.0 | 50.0 | 95.0 | 95.0 |  |  |  |  |  |  |  |  |  |  |  |  |  |  |  |  |  |  |  |  |
| Kappa (95% CI [LL, UL]) | 1.00 (1.00, 1.00) | 1.00 (1.00, 1.00) | 0.10 (-0.09, 0.29) | 0.10 (-0.09, 0.29) | 0.10 (-0.09, 0.29) | 0.10 (-0.09, 0.29) | 0.10 (-0.09, 0.29) | 0 | 0.10 (-0.09, 0.29) | 0.20 (-0.14, 0.54) | 0 | 0.90 (0.71, 1.00) | 0.90 (0.71, 1.00) |  |  |  |  |  |  |  |  |  |  |  |  |  |  |  |  |  |  |  |  |
| **Elecsys N (n=2)** |  |  |  |  |  |  |  |  |  |  |  |  |  |  |  |  |  |  |  |  |  |  |  |  |  |  |  |  |  |  |  |  |  |
| Concordance (%) | 80.0 | 78.9 | 65.0 | 65.0 | 65.0 | 65.0 | 65.0 | 60.0 | 65.0 | 60.0 | 50.0 | 75.0 | 75.0 | 80.0 |  |  |  |  |  |  |  |  |  |  |  |  |  |  |  |  |  |  |  |
| Kappa (95% CI [LL, UL]) | 0.60 (0.28, 0.92) | 0.59 (0.26, 0.92) | -0.09 (-0.26, 0.07) | -0.09 (-0.26, 0.07) | -0.09 (-0.26, 0.07) | -0.09 (-0.26, 0.07) | -0.09 (-0.26, 0.07) | -0.18 (-0.38, 0.02) | -0.09 (-0.26, 0.07) | -0.05 (-0.46, 0.35) | -0.09 (-0.51, 0.33) | 0.48 (0.11, 0.85) | 0.48 (0.11, 0.85) | 0.60 (0.28, 0.92) |  |  |  |  |  |  |  |  |  |  |  |  |  |  |  |  |  |  |  |
| **Elecsys N (n=3)** |  |  |  |  |  |  |  |  |  |  |  |  |  |  |  |  |  |  |  |  |  |  |  |  |  |  |  |  |  |  |  |  |  |
| Concordance (%) | 85.0 | 84.2 | 70.0 | 70.0 | 70.0 | 70.0 | 70.0 | 65.0 | 70.0 | 65.0 | 55.0 | 80.0 | 80.0 | 85.0 | 95.0 |  |  |  |  |  |  |  |  |  |  |  |  |  |  |  |  |  |  |
| Kappa (95% CI [LL, UL]) | 0.70 (0.40, 1.00) | 0.69 (0.38, 1.00) | 0.18 (-0.14, 0.49) | 0.18 (-0.14, 0.49) | 0.18 (-0.14, 0.49) | 0.18 (-0.14, 0.49) | 0.18 (-0.14, 0.49) | 0.08 (-0.28, 0.43) | 0.18 (-0.14, 0.49) | 0.15 (-0.28, 0.57) | 0.04 (-0.40, 0.48) | 0.59 (0.24, 0.94) | 0.59 (0.24, 0.94) | 0.70 (0.40, 1.00) | 0.89 (0.67, 1.00) |  |  |  |  |  |  |  |  |  |  |  |  |  |  |  |  |  |  |
| **Elecsys N (n=4)** |  |  |  |  |  |  |  |  |  |  |  |  |  |  |  |  |  |  |  |  |  |  |  |  |  |  |  |  |  |  |  |  |  |
| Concordance (%) | 95.0 | 94.7 | 60.0 | 60.0 | 60.0 | 60.0 | 60.0 | 55.0 | 60.0 | 65.0 | 55.0 | 90.0 | 90.0 | 95.0 | 85.0 | 90.0 |  |  |  |  |  |  |  |  |  |  |  |  |  |  |  |  |  |
| Kappa (95% CI [LL, UL]) | 0.90 (0.71, 1.00) | 0.90 (0.70, 1.00) | 0.12 (-0.10, 0.35) | 0.12 (-0.10, 0.35) | 0.12 (-0.10, 0.35) | 0.12 (-0.10, 0.35) | 0.12 (-0.10, 0.35) | 0.02 (-0.27, 0.31) | 0.12 (-0.10, 0.35) | 0.26 (-0.12, 0.63) | 0.08 (-0.35, 0.52) | 0.80 (0.53, 1.00) | 0.80 (0.53, 1.00) | 0.90 (0.71, 1.00) | 0.69 (0.38, 1.00) | 0.79 (0.53, 1.00) |  |  |  |  |  |  |  |  |  |  |  |  |  |  |  |  |  |
| **VITROS (n=2)** |  |  |  |  |  |  |  |  |  |  |  |  |  |  |  |  |  |  |  |  |  |  |  |  |  |  |  |  |  |  |  |  |  |
| Concordance (%) | 50.0 | 52.6 | 5.0 | 5.0 | 5.0 | 5.0 | 5.0 | 10.0 | 5.0 | 20.0 | 40.0 | 45.0 | 45.0 | 50.0 | 30.0 | 35.0 | 45.0 |  |  |  |  |  |  |  |  |  |  |  |  |  |  |  |  |
| Kappa (95% CI [LL, UL]) | 0 | 0 | 0 | 0 | 0 | 0 | 0 | 0 | 0 | 0 | 0 | 0 | 0 | 0 | 0 | 0 | 0 |  |  |  |  |  |  |  |  |  |  |  |  |  |  |  |  |
| **VITROS (n=3)** |  |  |  |  |  |  |  |  |  |  |  |  |  |  |  |  |  |  |  |  |  |  |  |  |  |  |  |  |  |  |  |  |  |
| Concordance (%) | 50.0 | 52.6 | 5.0 | 5.0 | 5.0 | 5.0 | 5.0 | 10.0 | 5.0 | 20.0 | 40.0 | 45.0 | 45.0 | 50.0 | 30.0 | 35.0 | 45.0 | 100.0 |  |  |  |  |  |  |  |  |  |  |  |  |  |  |  |
| Kappa (95% CI [LL, UL]) | 0 | 0 | 0 | 0 | 0 | 0 | 0 | 0 | 0 | 0 | 0 | 0 | 0 | 0 | 0 | 0 | 0 | NA |  |  |  |  |  |  |  |  |  |  |  |  |  |  |  |
| **VITROS (n=4)** |  |  |  |  |  |  |  |  |  |  |  |  |  |  |  |  |  |  |  |  |  |  |  |  |  |  |  |  |  |  |  |  |  |
| Concordance (%) | 50.0 | 52.6 | 5.0 | 5.0 | 5.0 | 5.0 | 5.0 | 10.0 | 5.0 | 20.0 | 40. | 45.0 | 45.0 | 50.0 | 30.0 | 35.0 | 45.0 | 100.0 | 100.0 |  |  |  |  |  |  |  |  |  |  |  |  |  |  |
| Kappa (95% CI [LL, UL]) | 0 | 0 | 0 | 0 | 0 | 0 | 0 | 0 | 0 | 0 | 0 | 0 | 0 | 0 | 0 | 0 | 0 | NA | NA |  |  |  |  |  |  |  |  |  |  |  |  |  |  |
| **Architect (n=2)** |  |  |  |  |  |  |  |  |  |  |  |  |  |  |  |  |  |  |  |  |  |  |  |  |  |  |  |  |  |  |  |  |  |
| Concordance (%) | 55.0 | 52.6 | 90.0 | 90.0 | 90.0 | 90.0 | 90.0 | 85.0 | 90.0 | 85.0 | 65.0 | 60.0 | 60.0 | 55.0 | 65.0 | 60.0 | 60.0 | 5.0 | 5.0 | 5.0 |  |  |  |  |  |  |  |  |  |  |  |  |  |
| Kappa (95% CI [LL, UL]) | 0.10 (-0.09, 0.29) | 0.10 (-0.09, 0.28) | -0.05 (-0.13, 0.02) | -0.05 (-0.13, 0.02) | -0.05 (-0.13, 0.02) | -0.05 (-0.13, 0.02) | -0.05 (-0.13, 0.02) | -0.07 (-0.17, 0.03) | -0.05 (-0.13, 0.02) | 0.35 (-0.17, 0.86) | 0.15 (-0.12, 0.41) | 0.12 (-0.10, 0.35) | 0.12 (-0.10, 0.35) | 0.10 (-0.09, 0.29) | -0.09 (-0.26, 0.07) | -0.10 (-0.27, 0.08) | 0.12 (-0.10, 0.35) | 0 | 0 | 0 |  |  |  |  |  |  |  |  |  |  |  |  |  |
| **Architect (n=3)** |  |  |  |  |  |  |  |  |  |  |  |  |  |  |  |  |  |  |  |  |  |  |  |  |  |  |  |  |  |  |  |  |  |
| Concordance (%) | 55.0 | 52.6 | 90.0 | 90.0 | 90.0 | 90.0 | 90.0 | 85.0 | 90.0 | 85.0 | 65.0 | 60.0 | 60.0 | 55.0 | 65.0 | 60.0 | 60.0 | 5.0 | 5.0 | 5.0 | 100.0 |  |  |  |  |  |  |  |  |  |  |  |  |
| Kappa (95% CI [LL, UL]) | 0.10 (-0.09, 0.29) | 0.10 (-0.09, 0.28) | -0.05 (-0.13, 0.02) | -0.05 (-0.13, 0.02) | -0.05 (-0.13, 0.02) | -0.05 (-0.13, 0.02) | -0.05 (-0.13, 0.02) | -0.07 (-0.17, 0.03) | -0.05 (-0.13, 0.02) | 0.35 (-0.17, 0.86) | 0.15 (-0.12, 0.41) | 0.12 (-0.10, 0.35) | 0.12 (-0.10, 0.35) | 0.10 (-0.09, 0.29) | -0.09 (-0.26, 0.07) | -0.10 (-0.27, 0.08) | 0.12 (-0.10, 0.35) | 0 | 0 | 0 | 1.00 (1.00, 1.00) |  |  |  |  |  |  |  |  |  |  |  |  |
| **Architect (n=4)** |  |  |  |  |  |  |  |  |  |  |  |  |  |  |  |  |  |  |  |  |  |  |  |  |  |  |  |  |  |  |  |  |  |
| Concordance (%) | 60.0 | 57.9 | 85.0 | 85.0 | 85.0 | 85.0 | 85.0 | 80.0 | 85.0 | 80.0 | 70.0 | 65.0 | 65.0 | 60.0 | 70.0 | 65.0 | 65.0 | 10.0 | 10.0 | 10.0 | 95.0 | 95.0 |  |  |  |  |  |  |  |  |  |  |  |
| Kappa (95% CI [LL, UL]) | 0.19 (-0.06, 0.46) | 0.19 (-0.06, 0.44) | -0.07 (-0.17, 0.03) | -0.07 (-0.17, 0.03) | -0.07 (-0.17, 0.03) | -0.07 (-0.17, 0.03) | -0.07 (-0.17, 0.03) | -0.11 (-0.22, -0.003) | -0.07 (-0.17, 0.03) | 0.23 (-0.29, 0.75) | 0.29 (-0.05, 0.62) | 0.24 (-0.06, 0.53) | 0.24 (-0.06, 0.53) | 0.20 (-0.06, 0.46) | 0.12 (-0.28, 0.52) | 0.08 (-0.28, 0.43) | 0.24 (-0.06, 0.53) | 0 | 0 | 0 | 0.64 (0.01, 1.00) | 0.64 (0.01, 1.00) |  |  |  |  |  |  |  |  |  |  |  |
| **GSP/DELFIA (n=4)** |  |  |  |  |  |  |  |  |  |  |  |  |  |  |  |  |  |  |  |  |  |  |  |  |  |  |  |  |  |  |  |  |  |
| Concordance (%) | 100.0 | 100.0 | 55.0 | 55.0 | 55.0 | 55.0 | 55.0 | 50.0 | 55.0 | 60.0 | 50.0 | 95.0 | 95.0 | 100.0 | 80.0 | 85.0 | 95.0 | 50.0 | 50.0 | 50.0 | 55.0 | 55.0 | 60.0 |  |  |  |  |  |  |  |  |  |  |
| Kappa (95% CI [LL, UL]) | 1.00 (1.00, 1.00) | 1.00 (1.00, 1.00) | 0.10 (-0.09, 0.29) | 0.10 (-0.09, 0.29) | 0.10 (-0.09, 0.29) | 0.10 (-0.09, 0.29) | 0.10 (-0.09, 0.29) | 0 | 0.10 (-0.09, 0.29) | 0.20 (-0.14, 0.54) | 0 | 0.90 (0.71, 1.00) | 0.90 (0.71, 1.00) | 1.00 (1.00, 1.00) | 0.60 (0.28, 0.92) | 0.70 (0.40, 1.00) | 0.90 (0.71, 1.00) | 0 | 0 | 0 | 0.10 (-0.09, 0.29) | 0.10 (-0.09, 0.29) | 0.20 (-0.06, 0.46) |  |  |  |  |  |  |  |  |  |  |
| **In-house S (U of T)** |  |  |  |  |  |  |  |  |  |  |  |  |  |  |  |  |  |  |  |  |  |  |  |  |  |  |  |  |  |  |  |  |  |
| Concordance (%) | 100.0 | 100.0 | 55.0 | 55.0 | 55.0 | 55.0 | 55.0 | 50.0 | 55.0 | 60.0 | 50.0 | 95.0 | 95.0 | 100.0 | 80.0 | 85.0 | 95.0 | 50.0 | 50.0 | 50.0 | 55.0 | 55.0 | 60.0 | 100.0 |  |  |  |  |  |  |  |  |  |
| Kappa (95% CI [LL, UL]) | 1.00 (1.00, 1.00) | 1.00 (1.00, 1.00) | 0.10 (-0.09, 0.29) | 0.10 (-0.09, 0.29) | 0.10 (-0.09, 0.29) | 0.10 (-0.09, 0.29) | 0.10 (-0.09, 0.29) | 0 | 0.10 (-0.09, 0.29) | 0.20 (-0.14, 0.54) | 0 | 0.90 (0.71, 1.00) | 0.90 (0.71, 1.00) | 1.00 (1.00, 1.00) | 0.60 (0.28, 0.92) | 0.70 (0.40, 1.00) | 0.90 (0.71, 1.00) | 0 | 0 | 0 | 0.10 (-0.09, 0.29) | 0.10 (-0.09, 0.29) | 0.20 (-0.06, 0.46) | 1.00 (1.00, 1.00) |  |  |  |  |  |  |  |  |  |
| **In-house RBD (U of T)** |  |  |  |  |  |  |  |  |  |  |  |  |  |  |  |  |  |  |  |  |  |  |  |  |  |  |  |  |  |  |  |  |  |
| Concordance (%) | 100.0 | 100.0 | 55.0 | 55.0 | 55.0 | 55.0 | 55.0 | 50.0 | 55.0 | 60.0 | 50.0 | 95.0 | 95.0 | 100.0 | 80.0 | 85.0 | 95.0 | 50.0 | 50.0 | 50.0 | 55.0 | 55.0 | 60.0 | 100.0 | 100.0 |  |  |  |  |  |  |  |  |
| Kappa (95% CI [LL, UL]) | 1.00 (1.00, 1.00) | 1.00 (1.00, 1.00) | 0.10 (-0.09, 0.29) | 0.10 (-0.09, 0.29) | 0.10 (-0.09, 0.29) | 0.10 (-0.09, 0.29) | 0.10 (-0.09, 0.29) | 0 | 0.10 (-0.09, 0.29) | 0.20 (-0.14, 0.54) | 0 | 0.90 (0.71, 1.00) | 0.90 (0.71, 1.00) | 1.00 (1.00, 1.00) | 0.60 (0.28, 0.92) | 0.70 (0.40, 1.00) | 0.90 (0.71, 1.00) | 0 | 0 | 0 | 0.10 (-0.09, 0.29) | 0.10 (-0.09, 0.29) | 0.20 (-0.06, 0.46) | 1.00 (1.00, 1.00) | 1.00 (1.00, 1.00) |  |  |  |  |  |  |  |  |
| **In-house N (U of T)** |  |  |  |  |  |  |  |  |  |  |  |  |  |  |  |  |  |  |  |  |  |  |  |  |  |  |  |  |  |  |  |  |  |
| Concordance (%) | 70.0 | 68.4 | 85.0 | 85.0 | 85.0 | 85.0 | 85.0 | 80.0 | 85.0 | 80.0 | 70.0 | 75.0 | 75.0 | 70.0 | 80.0 | 85.0 | 75.0 | 20.0 | 20.0 | 20.0 | 75.0 | 75.0 | 80.0 | 70.0 | 70.0 | 70.0 |  |  |  |  |  |  |  |
| Kappa (95% CI [LL, UL]) | 0.40 (0.08, 0.72) | 0.39 (0.07, 0.71) | 0.35 (-0.17, 0.86) | 0.35 (-0.17, 0.86) | 0.35 (-0.17, 0.86) | 0.35 (-0.17, 0.86) | 0.35 (-0.17, 0.86) | 0.23 (-0.29, 0.75) | 0.35 (-0.17, 0.86) | 0.38 (-0.12, 0.87) | 0.32 (-0.08, 0.71) | 0.47 (0.13, 0.81) | 0.47 (0.13, 0.81) | 0.40 (0.08, 0.72) | 0.47 (0.04, 0.90) | 0.63 (0.28, 0.99) | 0.47 (0.13, 0.81) | 0 | 0 | 0 | -0.09 (-0.23, 0.06) | -0.09 (-0.23, 0.06) | 0.23 (-0.29, 0.75) | 0.40 (0.08, 0.72) | 0.40 (0.08, 0.72) | 0.40 (0.08, 0.72) |  |  |  |  |  |  |  |
| **In-house S, poly (U of O)** |  |  |  |  |  |  |  |  |  |  |  |  |  |  |  |  |  |  |  |  |  |  |  |  |  |  |  |  |  |  |  |  |  |
| Concordance (%) | 100.0 | 100.0 | 55.0 | 55.0 | 55.0 | 55.0 | 55.0 | 50.0 | 55.0 | 60.0 | 50.0 | 95.0 | 95.0 | 100.0 | 80.0 | 85.0 | 95.0 | 50.0 | 50.0 | 50.0 | 55.0 | 55.0 | 60.0 | 100.0 | 100.0 | 100.0 | 70.0 |  |  |  |  |  |  |
| Kappa (95% CI [LL, UL]) | 1.00 (1.00, 1.00) | 1.00 (1.00, 1.00) | 0.10 (-0.09, 0.29) | 0.10 (-0.09, 0.29) | 0.10 (-0.09, 0.29) | 0.10 (-0.09, 0.29) | 0.10 (-0.09, 0.29) | 0 | 0.10 (-0.09, 0.29) | 0.20 (-0.14, 0.54) | 0 | 0.90 (0.71, 1.00) | 0.90 (0.71, 1.00) | 1.00 (1.00, 1.00) | 0.60 (0.28, 0.92) | 0.70 (0.40, 1.00) | 0.90 (0.71, 1.00) | 0 | 0 | 0 | 0.10 (-0.09, 0.29) | 0.10 (-0.09, 0.29) | 0.20 (-0.06, 0.46) | 1.00 (1.00, 1.00) | 1.00 (1.00, 1.00) | 1.00 (1.00, 1.00) | 0.40 (0.08, 0.72) |  |  |  |  |  |  |
| **In-house RBD, poly (U of O)** |  |  |  |  |  |  |  |  |  |  |  |  |  |  |  |  |  |  |  |  |  |  |  |  |  |  |  |  |  |  |  |  |  |
| Concordance (%) | 100.0 | 100.0 | 55.0 | 55.0 | 55.0 | 55.0 | 55.0 | 50.0 | 55.0 | 60.0 | 50.0 | 95.0 | 95.0 | 100.0 | 80.0 | 85.0 | 95.0 | 50.0 | 50.0 | 50.0 | 55.0 | 55.0 | 60.0 | 100.0 | 100.0 | 100.0 | 70.0 | 100.0 |  |  |  |  |  |
| Kappa (95% CI [LL, UL]) | 1.00 (1.00, 1.00) | 1.00 (1.00, 1.00) | 0.10 (-0.09, 0.29) | 0.10 (-0.09, 0.29) | 0.10 (-0.09, 0.29) | 0.10 (-0.09, 0.29) | 0.10 (-0.09, 0.29) | 0 | 0.10 (-0.09, 0.29) | 0.20 (-0.14, 0.54) | 0 | 0.90 (0.71, 1.00) | 0.90 (0.71, 1.00) | 1.00 (1.00, 1.00) | 0.60 (0.28, 0.92) | 0.70 (0.40, 1.00) | 0.90 (0.71, 1.00) | 0 | 0 | 0 | 0.10 (-0.09, 0.29) | 0.10 (-0.09, 0.29) | 0.20 (-0.06, 0.46) | 1.00 (1.00, 1.00) | 1.00 (1.00, 1.00) | 1.00 (1.00, 1.00) | 0.40 (0.08, 0.72) | 1.00 (1.00, 1.00) |  |  |  |  |  |
| **In-house N, poly (U of O)** |  |  |  |  |  |  |  |  |  |  |  |  |  |  |  |  |  |  |  |  |  |  |  |  |  |  |  |  |  |  |  |  |  |
| Concordance (%) | 100.0 | 100.0 | 55.0 | 55.0 | 55.0 | 55.0 | 55.0 | 50.0 | 55.0 | 60.0 | 50.0 | 95.0 | 95.0 | 100.0 | 80.0 | 85.0 | 95.0 | 50.0 | 50.0 | 50.0 | 55.0 | 55.0 | 60.0 | 100.0 | 100.0 | 100.0 | 70.0 | 100.0 | 100.0 |  |  |  |  |
| Kappa (95% CI [LL, UL]) | 1.00 (1.00, 1.00) | 1.00 (1.00, 1.00) | 0.10 (-0.09, 0.29) | 0.10 (-0.09, 0.29) | 0.10 (-0.09, 0.29) | 0.10 (-0.09, 0.29) | 0.10 (-0.09, 0.29) | 0 | 0.10 (-0.09, 0.29) | 0.20 (-0.14, 0.54) | 0 | 0.90 (0.71, 1.00) | 0.90 (0.71, 1.00) | 1.00 (1.00, 1.00) | 0.60 (0.28, 0.92) | 0.70 (0.40, 1.00) | 0.90 (0.71, 1.00) | 0 | 0 | 0 | 0.10 (-0.09, 0.29) | 0.10 (-0.09, 0.29) | 0.20 (-0.06, 0.46) | 1.00 (1.00, 1.00) | 1.00 (1.00, 1.00) | 1.00 (1.00, 1.00) | 0.40 (0.08, 0.72) | 1.00 (1.00, 1.00) | 1.00 (1.00, 1.00) |  |  |  |  |
| **In-house S, mono (U of O)** |  |  |  |  |  |  |  |  |  |  |  |  |  |  |  |  |  |  |  |  |  |  |  |  |  |  |  |  |  |  |  |  |  |
| Concordance (%) | 100.0 | 100.0 | 55.0 | 55.0 | 55.0 | 55.0 | 55.0 | 50.0 | 55.0 | 60.0 | 50.0 | 95.0 | 95.0 | 100.0 | 80.0 | 85.0 | 95.0 | 50.0 | 50.0 | 50.0 | 55.0 | 55.0 | 60.0 | 100.0 | 100.0 | 100.0 | 70.0 | 100.0 | 100.0 | 100.0 |  |  |  |
| Kappa (95% CI [LL, UL]) | 1.00 (1.00, 1.00) | 1.00 (1.00, 1.00) | 0.10 (-0.09, 0.29) | 0.10 (-0.09, 0.29) | 0.10 (-0.09, 0.29) | 0.10 (-0.09, 0.29) | 0.10 (-0.09, 0.29) | 0 | 0.10 (-0.09, 0.29) | 0.20 (-0.14, 0.54) | 0 | 0.90 (0.71, 1.00) | 0.90 (0.71, 1.00) | 1.00 (1.00, 1.00) | 0.60 (0.28, 0.92) | 0.70 (0.40, 1.00) | 0.90 (0.71, 1.00) | 0 | 0 | 0 | 0.10 (-0.09, 0.29) | 0.10 (-0.09, 0.29) | 0.20 (-0.06, 0.46) | 1.00 (1.00, 1.00) | 1.00 (1.00, 1.00) | 1.00 (1.00, 1.00) | 0.40 (0.08, 0.72) | 1.00 (1.00, 1.00) | 1.00 (1.00, 1.00) | 1.00 (1.00, 1.00) |  |  |  |
| **In-house RBD, mono (U of O)** |  |  |  |  |  |  |  |  |  |  |  |  |  |  |  |  |  |  |  |  |  |  |  |  |  |  |  |  |  |  |  |  |  |
| Concordance (%) | 100.0 | 100.0 | 55.0 | 55.0 | 55.0 | 55.0 | 55.0 | 50.0 | 55.0 | 60.0 | 50.0 | 95.0 | 95.0 | 100.0 | 80.0 | 85.0 | 95.0 | 50.0 | 50.0 | 50.0 | 55.0 | 55.0 | 60.0 | 100.0 | 100.0 | 100.0 | 70.0 | 100.0 | 100.0 | 100.0 | 100.0 |  |  |
| Kappa (95% CI [LL, UL]) | 1.00 (1.00, 1.00) | 1.00 (1.00, 1.00) | 0.10 (-0.09, 0.29) | 0.10 (-0.09, 0.29) | 0.10 (-0.09, 0.29) | 0.10 (-0.09, 0.29) | 0.10 (-0.09, 0.29) | 0 | 0.10 (-0.09, 0.29) | 0.20 (-0.14, 0.54) | 0 | 0.90 (0.71, 1.00) | 0.90 (0.71, 1.00) | 1.00 (1.00, 1.00) | 0.60 (0.28, 0.92) | 0.70 (0.40, 1.00) | 0.90 (0.71, 1.00) | 0 | 0 | 0 | 0.10 (-0.09, 0.29) | 0.10 (-0.09, 0.29) | 0.20 (-0.06, 0.46) | 1.00 (1.00, 1.00) | 1.00 (1.00, 1.00) | 1.00 (1.00, 1.00) | 0.40 (0.08, 0.72) | 1.00 (1.00, 1.00) | 1.00 (1.00, 1.00) | 1.00 (1.00, 1.00) | 1.00 (1.00, 1.00) |  |  |
| **In-house N, mono (U of O)** |  |  |  |  |  |  |  |  |  |  |  |  |  |  |  |  |  |  |  |  |  |  |  |  |  |  |  |  |  |  |  |  |  |
| Concordance (%) | 100.0 | 100.0 | 55.0 | 55.0 | 55.0 | 55.0 | 55.0 | 50.0 | 55.0 | 60.0 | 50.0 | 95.0 | 95.0 | 100.0 | 80.0 | 85.0 | 95.0 | 50.0 | 50.0 | 50.0 | 55.0 | 55.0 | 60.0 | 100.0 | 100.0 | 100.0 | 70.0 | 100.0 | 100.0 | 100.0 | 100.0 | 100.0 |  |
| Kappa (95% CI [LL, UL]) | 1.00 (1.00, 1.00) | 1.00 (1.00, 1.00) | 0.10 (-0.09, 0.29) | 0.10 (-0.09, 0.29) | 0.10 (-0.09, 0.29) | 0.10 (-0.09, 0.29) | 0.10 (-0.09, 0.29) | 0 | 0.10 (-0.09, 0.29) | 0.20 (-0.14, 0.54) | 0 | 0.90 (0.71, 1.00) | 0.90 (0.71, 1.00) | 1.00 (1.00, 1.00) | 0.60 (0.28, 0.92) | 0.70 (0.40, 1.00) | 0.90 (0.71, 1.00) | 0 | 0 | 0 | 0.10 (-0.09, 0.29) | 0.10 (-0.09, 0.29) | 0.20 (-0.06, 0.46) | 1.00 (1.00, 1.00) | 1.00 (1.00, 1.00) | 1.00 (1.00, 1.00) | 0.40 (0.08, 0.72) | 1.00 (1.00, 1.00) | 1.00 (1.00, 1.00) | 1.00 (1.00, 1.00) | 1.00 (1.00, 1.00) | 1.00 (1.00, 1.00) |  |
