# Supplementary material for: Dried blood spot specimens for SARS-CoV-2 antibody testing: A multi-site, multi-assay comparison
Source: PLoS One. 2021 Dec 7;16(12):e0261003. doi: 10.1371/journal.pone.0261003 (PMC8651133; doi:10.1371/journal.pone.0261003)
Supplement: S2 Table — (DOCX) [file pone.0261003.s006.docx]

**S2 Table**

| **Assay** | **Output** | **Target** | **DBS punches (n=)** | **Sample ID, SARS-CoV-2 negative** | **Result** | **Sample ID, SARS-CoV-2 positive** | **Result** |
| --- | --- | --- | --- | --- | --- | --- | --- |
| **Anti-SARS-CoV-2 ELISA (EUROIMMUN)** | OD ratio | Spike (S1) | 2 | J7 | 0.20 | 20-1955 | 2.51 |
| **Anti-SARS-CoV-2 ELISA (EUROIMMUN)** | OD ratio | Spike (S1) | 2 | J30 | 0.12 | 20-1954 | 1.92 |
| **Anti-SARS-CoV-2 ELISA (EUROIMMUN)** | OD ratio | Spike (S1) | 2 | J19 | 0.15 | 20-1952 | 1.32 |
| **Anti-SARS-CoV-2 ELISA (EUROIMMUN)** | OD ratio | Spike (S1) | 2 | J15 | 0.15 | 20-1887 | 3.93 |
| **Anti-SARS-CoV-2 ELISA (EUROIMMUN)** | OD ratio | Spike (S1) | 2 | J12 | 0.12 | 20-1886 | 3.95 |
| **Anti-SARS-CoV-2 ELISA (EUROIMMUN)** | OD ratio | Spike (S1) | 2 | J11 | 0.30 | 20-1885 | 3.14 |
| **Anti-SARS-CoV-2 ELISA (EUROIMMUN)** | OD ratio | Spike (S1) | 2 | J1 | 0.20 | 20-1882 | 4.29 |
| **Anti-SARS-CoV-2 ELISA (EUROIMMUN)** | OD ratio | Spike (S1) | 2 | D9 | 0.20 | 20-1879 | 9.10 |
| **Anti-SARS-CoV-2 ELISA (EUROIMMUN)** | OD ratio | Spike (S1) | 2 | D17 | 0.16 | 20-1878 | 4.93 |
| **Anti-SARS-CoV-2 ELISA (EUROIMMUN)** | OD ratio | Spike (S1) | 2 | D142 | 0.31 | 20-1877 | 4.11 |
| **Platelia SARS-CoV-2 (Bio-Rad)** | OD ratio | Nucleocapsid | 2 | J7 | 0.46 | 20-1955 | 1.88 |
| **Platelia SARS-CoV-2 (Bio-Rad)** | OD ratio | Nucleocapsid | 2 | J30 | 0.29 | 20-1954 | 1.31 |
| **Platelia SARS-CoV-2 (Bio-Rad)** | OD ratio | Nucleocapsid | 2 | J19 | 0.43 | 20-1952 | 1.15 |
| **Platelia SARS-CoV-2 (Bio-Rad)** | OD ratio | Nucleocapsid | 2 | J15 | 0.63 | 20-1887 | 4.00 |
| **Platelia SARS-CoV-2 (Bio-Rad)** | OD ratio | Nucleocapsid | 2 | J12 | 0.54 | 20-1886 | 2.12 |
| **Platelia SARS-CoV-2 (Bio-Rad)** | OD ratio | Nucleocapsid | 2 | J11 | 0.43 | 20-1885 | 3.12 |
| **Platelia SARS-CoV-2 (Bio-Rad)** | OD ratio | Nucleocapsid | 2 | J1 | 0.29 | 20-1882 | 3.44 |
| **Platelia SARS-CoV-2 (Bio-Rad)** | OD ratio | Nucleocapsid | 2 | D9 | 0.49 | 20-1879 | 4.00 |
| **Platelia SARS-CoV-2 (Bio-Rad)** | OD ratio | Nucleocapsid | 2 | D17 | 0.49 | 20-1878 | 4.00 |
| **Platelia SARS-CoV-2 (Bio-Rad)** | OD ratio | Nucleocapsid | 2 | D142 | 0.81 | 20-1877 | 4.00 |
| **LIASON SARS-CoV-2 (DiaSorin)** | AU/mL | Spike (S1, S2) | 2 | J7 | 3.80 | 20-1955 | 3.80 |
| **LIASON SARS-CoV-2 (DiaSorin)** | AU/mL | Spike (S1, S2) | 2 | J30 | 3.80 | 20-1954 | 3.80 |
| **LIASON SARS-CoV-2 (DiaSorin)** | AU/mL | Spike (S1, S2) | 2 | J19 | 3.80 | 20-1952 | 3.80 |
| **LIASON SARS-CoV-2 (DiaSorin)** | AU/mL | Spike (S1, S2) | 2 | J15 | 3.80 | 20-1887 | 3.80 |
| **LIASON SARS-CoV-2 (DiaSorin)** | AU/mL | Spike (S1, S2) | 2 | J12 | 3.80 | 20-1886 | 3.80 |
| **LIASON SARS-CoV-2 (DiaSorin)** | AU/mL | Spike (S1, S2) | 2 | J11 | 3.80 | 20-1885 | 8.27 |
| **LIASON SARS-CoV-2 (DiaSorin)** | AU/mL | Spike (S1, S2) | 2 | J1 | 3.80 | 20-1882 | 3.80 |
| **LIASON SARS-CoV-2 (DiaSorin)** | AU/mL | Spike (S1, S2) | 2 | D9 | 3.80 | 20-1879 | 37.70 |
| **LIASON SARS-CoV-2 (DiaSorin)** | AU/mL | Spike (S1, S2) | 2 | D17 | 3.80 | 20-1878 | 3.80 |
| **LIASON SARS-CoV-2 (DiaSorin)** | AU/mL | Spike (S1, S2) | 2 | D142 | 3.80 | 20-1877 | 3.80 |
| **LIASON SARS-CoV-2 (DiaSorin)** | AU/mL | Spike (S1, S2) | 3 | J7 | 3.80 | 20-1955 | 3.80 |
| **LIASON SARS-CoV-2 (DiaSorin)** | AU/mL | Spike (S1, S2) | 3 | J30 | 3.80 | 20-1954 | 3.80 |
| **LIASON SARS-CoV-2 (DiaSorin)** | AU/mL | Spike (S1, S2) | 3 | J19 | 3.80 | 20-1952 | 3.80 |
| **LIASON SARS-CoV-2 (DiaSorin)** | AU/mL | Spike (S1, S2) | 3 | J15 | 3.80 | 20-1887 | 3.80 |
| **LIASON SARS-CoV-2 (DiaSorin)** | AU/mL | Spike (S1, S2) | 3 | J12 | 3.80 | 20-1886 | 3.80 |
| **LIASON SARS-CoV-2 (DiaSorin)** | AU/mL | Spike (S1, S2) | 3 | J11 | 3.80 | 20-1885 | 9.28 |
| **LIASON SARS-CoV-2 (DiaSorin)** | AU/mL | Spike (S1, S2) | 3 | J1 | 3.80 | 20-1882 | 3.80 |
| **LIASON SARS-CoV-2 (DiaSorin)** | AU/mL | Spike (S1, S2) | 3 | D9 | 3.80 | 20-1879 | 39.90 |
| **LIASON SARS-CoV-2 (DiaSorin)** | AU/mL | Spike (S1, S2) | 3 | D17 | 3.80 | 20-1878 | 3.80 |
| **LIASON SARS-CoV-2 (DiaSorin)** | AU/mL | Spike (S1, S2) | 3 | D142 | 3.80 | 20-1877 | 3.80 |
| **LIASON SARS-CoV-2 (DiaSorin)** | AU/mL | Spike (S1, S2) | 4 | J7 | 3.80 | 20-1955 | 3.80 |
| **LIASON SARS-CoV-2 (DiaSorin)** | AU/mL | Spike (S1, S2) | 4 | J30 | 3.80 | 20-1954 | 3.80 |
| **LIASON SARS-CoV-2 (DiaSorin)** | AU/mL | Spike (S1, S2) | 4 | J19 | 3.80 | 20-1952 | 3.80 |
| **LIASON SARS-CoV-2 (DiaSorin)** | AU/mL | Spike (S1, S2) | 4 | J15 | 3.80 | 20-1887 | 3.80 |
| **LIASON SARS-CoV-2 (DiaSorin)** | AU/mL | Spike (S1, S2) | 4 | J12 | 3.80 | 20-1886 | 4.21 |
| **LIASON SARS-CoV-2 (DiaSorin)** | AU/mL | Spike (S1, S2) | 4 | J11 | 3.80 | 20-1885 | 12.00 |
| **LIASON SARS-CoV-2 (DiaSorin)** | AU/mL | Spike (S1, S2) | 4 | J1 | 3.80 | 20-1882 | 3.80 |
| **LIASON SARS-CoV-2 (DiaSorin)** | AU/mL | Spike (S1, S2) | 4 | D9 | 3.80 | 20-1879 | 51.60 |
| **LIASON SARS-CoV-2 (DiaSorin)** | AU/mL | Spike (S1, S2) | 4 | D17 | 3.80 | 20-1878 | 3.80 |
| **LIASON SARS-CoV-2 (DiaSorin)** | AU/mL | Spike (S1, S2) | 4 | D142 | 3.80 | 20-1877 | 3.80 |
| **SARS-CoV-2 COV2G (Siemens)** | AU/mL | Spike (S1) | 2 | J7 | 0.55 | 20-1955 | 0.19 |
| **SARS-CoV-2 COV2G (Siemens)** | AU/mL | Spike (S1) | 2 | J30 | 0.38 | 20-1954 | 0.23 |
| **SARS-CoV-2 COV2G (Siemens)** | AU/mL | Spike (S1) | 2 | J19 | 0.39 | 20-1952 | 0.24 |
| **SARS-CoV-2 COV2G (Siemens)** | AU/mL | Spike (S1) | 2 | J15 | 0.21 | 20-1887 | 0.23 |
| **SARS-CoV-2 COV2G (Siemens)** | AU/mL | Spike (S1) | 2 | J12 | 0.59 | 20-1886 | 0.31 |
| **SARS-CoV-2 COV2G (Siemens)** | AU/mL | Spike (S1) | 2 | J11 | 0.29 | 20-1885 | 0.34 |
| **SARS-CoV-2 COV2G (Siemens)** | AU/mL | Spike (S1) | 2 | J1 | 0.22 | 20-1882 | 0.31 |
| **SARS-CoV-2 COV2G (Siemens)** | AU/mL | Spike (S1) | 2 | D9 | 0.26 | 20-1879 | 2.55 |
| **SARS-CoV-2 COV2G (Siemens)** | AU/mL | Spike (S1) | 2 | D17 | 0.47 | 20-1878 | 0.31 |
| **SARS-CoV-2 COV2G (Siemens)** | AU/mL | Spike (S1) | 2 | D142 | 0.30 | 20-1877 | 0.36 |
| **SARS-CoV-2 COV2G (Siemens)** | AU/mL | Spike (S1) | 3 | J7 | 0.81 | 20-1955 | 0.31 |
| **SARS-CoV-2 COV2G (Siemens)** | AU/mL | Spike (S1) | 3 | J30 | 0.46 | 20-1954 | 0.34 |
| **SARS-CoV-2 COV2G (Siemens)** | AU/mL | Spike (S1) | 3 | J19 | 0.66 | 20-1952 | 0.46 |
| **SARS-CoV-2 COV2G (Siemens)** | AU/mL | Spike (S1) | 3 | J15 | 0.29 | 20-1887 | 0.31 |
| **SARS-CoV-2 COV2G (Siemens)** | AU/mL | Spike (S1) | 3 | J12 | 0.86 | 20-1886 | 0.44 |
| **SARS-CoV-2 COV2G (Siemens)** | AU/mL | Spike (S1) | 3 | J11 | 0.60 | 20-1885 | 0.39 |
| **SARS-CoV-2 COV2G (Siemens)** | AU/mL | Spike (S1) | 3 | J1 | 0.46 | 20-1882 | 0.47 |
| **SARS-CoV-2 COV2G (Siemens)** | AU/mL | Spike (S1) | 3 | D9 | 0.34 | 20-1879 | 3.04 |
| **SARS-CoV-2 COV2G (Siemens)** | AU/mL | Spike (S1) | 3 | D17 | 0.42 | 20-1878 | 0.38 |
| **SARS-CoV-2 COV2G (Siemens)** | AU/mL | Spike (S1) | 3 | D142 | 0.51 | 20-1877 | 0.50 |
| **SARS-CoV-2 COV2G (Siemens)** | AU/mL | Spike (S1) | 4 | J7 | 1.12 | 20-1955 | 0.38 |
| **SARS-CoV-2 COV2G (Siemens)** | AU/mL | Spike (S1) | 4 | J30 | 0.62 | 20-1954 | 0.41 |
| **SARS-CoV-2 COV2G (Siemens)** | AU/mL | Spike (S1) | 4 | J19 | 0.80 | 20-1952 | 0.48 |
| **SARS-CoV-2 COV2G (Siemens)** | AU/mL | Spike (S1) | 4 | J15 | 0.37 | 20-1887 | 0.43 |
| **SARS-CoV-2 COV2G (Siemens)** | AU/mL | Spike (S1) | 4 | J12 | 1.00 | 20-1886 | 0.43 |
| **SARS-CoV-2 COV2G (Siemens)** | AU/mL | Spike (S1) | 4 | J11 | 0.68 | 20-1885 | 0.57 |
| **SARS-CoV-2 COV2G (Siemens)** | AU/mL | Spike (S1) | 4 | J1 | 0.40 | 20-1882 | 0.58 |
| **SARS-CoV-2 COV2G (Siemens)** | AU/mL | Spike (S1) | 4 | D9 | 0.46 | 20-1879 | 4.13 |
| **SARS-CoV-2 COV2G (Siemens)** | AU/mL | Spike (S1) | 4 | D17 | 0.57 | 20-1878 | 0.58 |
| **SARS-CoV-2 COV2G (Siemens)** | AU/mL | Spike (S1) | 4 | D142 | 0.63 | 20-1877 | 0.56 |
| **SARS-CoV-2 COV2T (Siemens)** | AU/mL | Spike (S1) | 2 | J7 | 0.90 | 20-1955 | 0.61 |
| **SARS-CoV-2 COV2T (Siemens)** | AU/mL | Spike (S1) | 2 | J30 | 0.44 | 20-1954 | 0.45 |
| **SARS-CoV-2 COV2T (Siemens)** | AU/mL | Spike (S1) | 2 | J19 | 0.48 | 20-1952 | 0.54 |
| **SARS-CoV-2 COV2T (Siemens)** | AU/mL | Spike (S1) | 2 | J15 | 0.41 | 20-1887 | 0.39 |
| **SARS-CoV-2 COV2T (Siemens)** | AU/mL | Spike (S1) | 2 | J12 | 0.81 | 20-1886 | 0.90 |
| **SARS-CoV-2 COV2T (Siemens)** | AU/mL | Spike (S1) | 2 | J11 | 0.77 | 20-1885 | 0.93 |
| **SARS-CoV-2 COV2T (Siemens)** | AU/mL | Spike (S1) | 2 | J1 | 0.76 | 20-1882 | 0.56 |
| **SARS-CoV-2 COV2T (Siemens)** | AU/mL | Spike (S1) | 2 | D9 | 0.54 | 20-1879 | 6.18 |
| **SARS-CoV-2 COV2T (Siemens)** | AU/mL | Spike (S1) | 2 | D17 | 0.42 | 20-1878 | 0.56 |
| **SARS-CoV-2 COV2T (Siemens)** | AU/mL | Spike (S1) | 2 | D142 | 0.80 | 20-1877 | 0.79 |
| **SARS-CoV-2 COV2T (Siemens)** | AU/mL | Spike (S1) | 3 | J7 | 1.08 | 20-1955 | 0.49 |
| **SARS-CoV-2 COV2T (Siemens)** | AU/mL | Spike (S1) | 3 | J30 | 0.50 | 20-1954 | 0.76 |
| **SARS-CoV-2 COV2T (Siemens)** | AU/mL | Spike (S1) | 3 | J19 | 0.54 | 20-1952 | 0.81 |
| **SARS-CoV-2 COV2T (Siemens)** | AU/mL | Spike (S1) | 3 | J15 | 0.46 | 20-1887 | 0.45 |
| **SARS-CoV-2 COV2T (Siemens)** | AU/mL | Spike (S1) | 3 | J12 | 0.97 | 20-1886 | 1.07 |
| **SARS-CoV-2 COV2T (Siemens)** | AU/mL | Spike (S1) | 3 | J11 | 0.88 | 20-1885 | 1.05 |
| **SARS-CoV-2 COV2T (Siemens)** | AU/mL | Spike (S1) | 3 | J1 | 0.92 | 20-1882 | 0.44 |
| **SARS-CoV-2 COV2T (Siemens)** | AU/mL | Spike (S1) | 3 | D9 | 0.56 | 20-1879 | 6.75 |
| **SARS-CoV-2 COV2T (Siemens)** | AU/mL | Spike (S1) | 3 | D17 | 0.41 | 20-1878 | 0.69 |
| **SARS-CoV-2 COV2T (Siemens)** | AU/mL | Spike (S1) | 3 | D142 | 0.78 | 20-1877 | 0.89 |
| **SARS-CoV-2 COV2T (Siemens)** | AU/mL | Spike (S1) | 4 | J7 | 0.87 | 20-1955 | 0.81 |
| **SARS-CoV-2 COV2T (Siemens)** | AU/mL | Spike (S1) | 4 | J30 | 0.56 | 20-1954 | 0.73 |
| **SARS-CoV-2 COV2T (Siemens)** | AU/mL | Spike (S1) | 4 | J19 | 1.01 | 20-1952 | 0.73 |
| **SARS-CoV-2 COV2T (Siemens)** | AU/mL | Spike (S1) | 4 | J15 | 0.58 | 20-1887 | 0.47 |
| **SARS-CoV-2 COV2T (Siemens)** | AU/mL | Spike (S1) | 4 | J12 | 1.24 | 20-1886 | 0.94 |
| **SARS-CoV-2 COV2T (Siemens)** | AU/mL | Spike (S1) | 4 | J11 | 0.58 | 20-1885 | 1.24 |
| **SARS-CoV-2 COV2T (Siemens)** | AU/mL | Spike (S1) | 4 | J1 | 1.00 | 20-1882 | 0.61 |
| **SARS-CoV-2 COV2T (Siemens)** | AU/mL | Spike (S1) | 4 | D9 | 0.79 | 20-1879 | 8.38 |
| **SARS-CoV-2 COV2T (Siemens)** | AU/mL | Spike (S1) | 4 | D17 | 0.42 | 20-1878 | 1.04 |
| **SARS-CoV-2 COV2T (Siemens)** | AU/mL | Spike (S1) | 4 | D142 | 1.25 | 20-1877 | 1.36 |
| **Elecsys quantitative Anti-SARS-CoV-2 (Elecsys spike; Roche)** | U/mL | Spike | 2 | J7 | 0.40 | 20-1955 | 1.19 |
| **Elecsys quantitative Anti-SARS-CoV-2 (Elecsys spike; Roche)** | U/mL | Spike | 2 | J30 | 0.40 | 20-1954 | 0.87 |
| **Elecsys quantitative Anti-SARS-CoV-2 (Elecsys spike; Roche)** | U/mL | Spike | 2 | J19 | 0.40 | 20-1952 | 1.48 |
| **Elecsys quantitative Anti-SARS-CoV-2 (Elecsys spike; Roche)** | U/mL | Spike | 2 | J15 | 0.40 | 20-1887 | 0.40 |
| **Elecsys quantitative Anti-SARS-CoV-2 (Elecsys spike; Roche)** | U/mL | Spike | 2 | J12 | 0.40 | 20-1886 | 3.40 |
| **Elecsys quantitative Anti-SARS-CoV-2 (Elecsys spike; Roche)** | U/mL | Spike | 2 | J11 | 0.40 | 20-1885 | 2.56 |
| **Elecsys quantitative Anti-SARS-CoV-2 (Elecsys spike; Roche)** | U/mL | Spike | 2 | J1 | 0.40 | 20-1882 | 1.71 |
| **Elecsys quantitative Anti-SARS-CoV-2 (Elecsys spike; Roche)** | U/mL | Spike | 2 | D9 | 0.40 | 20-1879 | 36.69 |
| **Elecsys quantitative Anti-SARS-CoV-2 (Elecsys spike; Roche)** | U/mL | Spike | 2 | D17 | 0.40 | 20-1878 | 1.41 |
| **Elecsys quantitative Anti-SARS-CoV-2 (Elecsys spike; Roche)** | U/mL | Spike | 2 | D142 | 0.40 | 20-1877 | 1.19 |
| **Elecsys quantitative Anti-SARS-CoV-2 (Elecsys spike; Roche)** | U/mL | Spike | 3 | J7 | 0.40 | 20-1955 | 1.60 |
| **Elecsys quantitative Anti-SARS-CoV-2 (Elecsys spike; Roche)** | U/mL | Spike | 3 | J30 | 0.40 | 20-1954 | 1.35 |
| **Elecsys quantitative Anti-SARS-CoV-2 (Elecsys spike; Roche)** | U/mL | Spike | 3 | J19 | 0.40 | 20-1952 | 2.33 |
| **Elecsys quantitative Anti-SARS-CoV-2 (Elecsys spike; Roche)** | U/mL | Spike | 3 | J15 | 0.40 | 20-1887 | 0.50 |
| **Elecsys quantitative Anti-SARS-CoV-2 (Elecsys spike; Roche)** | U/mL | Spike | 3 | J12 | 0.40 | 20-1886 | 4.49 |
| **Elecsys quantitative Anti-SARS-CoV-2 (Elecsys spike; Roche)** | U/mL | Spike | 3 | J11 | 0.40 | 20-1885 | 3.65 |
| **Elecsys quantitative Anti-SARS-CoV-2 (Elecsys spike; Roche)** | U/mL | Spike | 3 | J1 | 0.40 | 20-1882 | 2.35 |
| **Elecsys quantitative Anti-SARS-CoV-2 (Elecsys spike; Roche)** | U/mL | Spike | 3 | D9 | 0.40 | 20-1879 | 56.45 |
| **Elecsys quantitative Anti-SARS-CoV-2 (Elecsys spike; Roche)** | U/mL | Spike | 3 | D17 | 0.40 | 20-1878 | 1.72 |
| **Elecsys quantitative Anti-SARS-CoV-2 (Elecsys spike; Roche)** | U/mL | Spike | 3 | D142 | 0.40 | 20-1877 | 1.50 |
| **Elecsys quantitative Anti-SARS-CoV-2 (Elecsys spike; Roche)** | U/mL | Spike | 4 | J7 | 0.40 | 20-1955 | 1.66 |
| **Elecsys quantitative Anti-SARS-CoV-2 (Elecsys spike; Roche)** | U/mL | Spike | 4 | J30 | 0.40 | 20-1954 | 1.43 |
| **Elecsys quantitative Anti-SARS-CoV-2 (Elecsys spike; Roche)** | U/mL | Spike | 4 | J19 | 0.40 | 20-1952 | 2.65 |
| **Elecsys quantitative Anti-SARS-CoV-2 (Elecsys spike; Roche)** | U/mL | Spike | 4 | J15 | 0.40 | 20-1887 | 0.65 |
| **Elecsys quantitative Anti-SARS-CoV-2 (Elecsys spike; Roche)** | U/mL | Spike | 4 | J12 | 0.40 | 20-1886 | 5.70 |
| **Elecsys quantitative Anti-SARS-CoV-2 (Elecsys spike; Roche)** | U/mL | Spike | 4 | J11 | 0.40 | 20-1885 | 3.93 |
| **Elecsys quantitative Anti-SARS-CoV-2 (Elecsys spike; Roche)** | U/mL | Spike | 4 | J1 | 0.40 | 20-1882 | 2.57 |
| **Elecsys quantitative Anti-SARS-CoV-2 (Elecsys spike; Roche)** | U/mL | Spike | 4 | D9 | 0.40 | 20-1879 | 70.47 |
| **Elecsys quantitative Anti-SARS-CoV-2 (Elecsys spike; Roche)** | U/mL | Spike | 4 | D17 | 0.40 | 20-1878 | 2.15 |
| **Elecsys quantitative Anti-SARS-CoV-2 (Elecsys spike; Roche)** | U/mL | Spike | 4 | D142 | 0.40 | 20-1877 | 1.82 |
| **Elecsys Anti-SARS-CoV-2 (Elecsys nucleocapsid; Roche)** | Cutoff index | Nucleocapsid | 2 | J7 | 0.05 | 20-1955 | 0.59 |
| **Elecsys Anti-SARS-CoV-2 (Elecsys nucleocapsid; Roche)** | Cutoff index | Nucleocapsid | 2 | J30 | 0.06 | 20-1954 | 0.62 |
| **Elecsys Anti-SARS-CoV-2 (Elecsys nucleocapsid; Roche)** | Cutoff index | Nucleocapsid | 2 | J19 | 0.05 | 20-1952 | 2.30 |
| **Elecsys Anti-SARS-CoV-2 (Elecsys nucleocapsid; Roche)** | Cutoff index | Nucleocapsid | 2 | J15 | 0.06 | 20-1887 | 1.09 |
| **Elecsys Anti-SARS-CoV-2 (Elecsys nucleocapsid; Roche)** | Cutoff index | Nucleocapsid | 2 | J12 | 0.05 | 20-1886 | 1.47 |
| **Elecsys Anti-SARS-CoV-2 (Elecsys nucleocapsid; Roche)** | Cutoff index | Nucleocapsid | 2 | J11 | 0.05 | 20-1885 | 0.72 |
| **Elecsys Anti-SARS-CoV-2 (Elecsys nucleocapsid; Roche)** | Cutoff index | Nucleocapsid | 2 | J1 | 0.05 | 20-1882 | 2.07 |
| **Elecsys Anti-SARS-CoV-2 (Elecsys nucleocapsid; Roche)** | Cutoff index | Nucleocapsid | 2 | D9 | 0.05 | 20-1879 | 0.85 |
| **Elecsys Anti-SARS-CoV-2 (Elecsys nucleocapsid; Roche)** | Cutoff index | Nucleocapsid | 2 | D17 | 0.05 | 20-1878 | 1.15 |
| **Elecsys Anti-SARS-CoV-2 (Elecsys nucleocapsid; Roche)** | Cutoff index | Nucleocapsid | 2 | D142 | 0.05 | 20-1877 | 2.31 |
| **Elecsys Anti-SARS-CoV-2 (Elecsys nucleocapsid; Roche)** | Cutoff index | Nucleocapsid | 3 | J7 | 0.05 | 20-1955 | 0.77 |
| **Elecsys Anti-SARS-CoV-2 (Elecsys nucleocapsid; Roche)** | Cutoff index | Nucleocapsid | 3 | J30 | 0.05 | 20-1954 | 0.78 |
| **Elecsys Anti-SARS-CoV-2 (Elecsys nucleocapsid; Roche)** | Cutoff index | Nucleocapsid | 3 | J19 | 0.05 | 20-1952 | 3.51 |
| **Elecsys Anti-SARS-CoV-2 (Elecsys nucleocapsid; Roche)** | Cutoff index | Nucleocapsid | 3 | J15 | 0.05 | 20-1887 | 1.25 |
| **Elecsys Anti-SARS-CoV-2 (Elecsys nucleocapsid; Roche)** | Cutoff index | Nucleocapsid | 3 | J12 | 0.05 | 20-1886 | 2.10 |
| **Elecsys Anti-SARS-CoV-2 (Elecsys nucleocapsid; Roche)** | Cutoff index | Nucleocapsid | 3 | J11 | 0.05 | 20-1885 | 0.96 |
| **Elecsys Anti-SARS-CoV-2 (Elecsys nucleocapsid; Roche)** | Cutoff index | Nucleocapsid | 3 | J1 | 0.05 | 20-1882 | 3.14 |
| **Elecsys Anti-SARS-CoV-2 (Elecsys nucleocapsid; Roche)** | Cutoff index | Nucleocapsid | 3 | D9 | 0.05 | 20-1879 | 1.20 |
| **Elecsys Anti-SARS-CoV-2 (Elecsys nucleocapsid; Roche)** | Cutoff index | Nucleocapsid | 3 | D17 | 0.05 | 20-1878 | 1.43 |
| **Elecsys Anti-SARS-CoV-2 (Elecsys nucleocapsid; Roche)** | Cutoff index | Nucleocapsid | 3 | D142 | 0.05 | 20-1877 | 3.39 |
| **Elecsys Anti-SARS-CoV-2 (Elecsys nucleocapsid; Roche)** | Cutoff index | Nucleocapsid | 4 | J7 | 0.05 | 20-1955 | 0.91 |
| **Elecsys Anti-SARS-CoV-2 (Elecsys nucleocapsid; Roche)** | Cutoff index | Nucleocapsid | 4 | J30 | 0.05 | 20-1954 | 1.10 |
| **Elecsys Anti-SARS-CoV-2 (Elecsys nucleocapsid; Roche)** | Cutoff index | Nucleocapsid | 4 | J19 | 0.05 | 20-1952 | 4.06 |
| **Elecsys Anti-SARS-CoV-2 (Elecsys nucleocapsid; Roche)** | Cutoff index | Nucleocapsid | 4 | J15 | 0.05 | 20-1887 | 1.88 |
| **Elecsys Anti-SARS-CoV-2 (Elecsys nucleocapsid; Roche)** | Cutoff index | Nucleocapsid | 4 | J12 | 0.05 | 20-1886 | 2.82 |
| **Elecsys Anti-SARS-CoV-2 (Elecsys nucleocapsid; Roche)** | Cutoff index | Nucleocapsid | 4 | J11 | 0.05 | 20-1885 | 1.31 |
| **Elecsys Anti-SARS-CoV-2 (Elecsys nucleocapsid; Roche)** | Cutoff index | Nucleocapsid | 4 | J1 | 0.05 | 20-1882 | 3.67 |
| **Elecsys Anti-SARS-CoV-2 (Elecsys nucleocapsid; Roche)** | Cutoff index | Nucleocapsid | 4 | D9 | 0.05 | 20-1879 | 1.58 |
| **Elecsys Anti-SARS-CoV-2 (Elecsys nucleocapsid; Roche)** | Cutoff index | Nucleocapsid | 4 | D17 | 0.05 | 20-1878 | 1.53 |
| **Elecsys Anti-SARS-CoV-2 (Elecsys nucleocapsid; Roche)** | Cutoff index | Nucleocapsid | 4 | D142 | 0.05 | 20-1877 | 4.95 |
| **VITROS Anti-SARS-CoV-2 (Ortho Clinical Diagnostics)** | S/Co | Spike (S1) | 2 | J7 | 3.23 | 20-1955 | 4.02 |
| **VITROS Anti-SARS-CoV-2 (Ortho Clinical Diagnostics)** | S/Co | Spike (S1) | 2 | J30 | 2.33 | 20-1954 | 3.44 |
| **VITROS Anti-SARS-CoV-2 (Ortho Clinical Diagnostics)** | S/Co | Spike (S1) | 2 | J19 | 2.71 | 20-1952 | 4.00 |
| **VITROS Anti-SARS-CoV-2 (Ortho Clinical Diagnostics)** | S/Co | Spike (S1) | 2 | J15 | 1.86 | 20-1887 | 2.39 |
| **VITROS Anti-SARS-CoV-2 (Ortho Clinical Diagnostics)** | S/Co | Spike (S1) | 2 | J12 | 2.68 | 20-1886 | 4.53 |
| **VITROS Anti-SARS-CoV-2 (Ortho Clinical Diagnostics)** | S/Co | Spike (S1) | 2 | J11 | 2.39 | 20-1885 | 5.53 |
| **VITROS Anti-SARS-CoV-2 (Ortho Clinical Diagnostics)** | S/Co | Spike (S1) | 2 | J1 | 2.86 | 20-1882 | 3.65 |
| **VITROS Anti-SARS-CoV-2 (Ortho Clinical Diagnostics)** | S/Co | Spike (S1) | 2 | D9 | 2.64 | 20-1879 | 37.90 |
| **VITROS Anti-SARS-CoV-2 (Ortho Clinical Diagnostics)** | S/Co | Spike (S1) | 2 | D17 | 2.40 | 20-1878 | 3.83 |
| **VITROS Anti-SARS-CoV-2 (Ortho Clinical Diagnostics)** | S/Co | Spike (S1) | 2 | D142 | 2.71 | 20-1877 | 3.54 |
| **VITROS Anti-SARS-CoV-2 (Ortho Clinical Diagnostics)** | S/Co | Spike (S1) | 3 | J7 | 4.75 | 20-1955 | 4.41 |
| **VITROS Anti-SARS-CoV-2 (Ortho Clinical Diagnostics)** | S/Co | Spike (S1) | 3 | J30 | 2.61 | 20-1954 | 3.93 |
| **VITROS Anti-SARS-CoV-2 (Ortho Clinical Diagnostics)** | S/Co | Spike (S1) | 3 | J19 | 2.57 | 20-1952 | 4.84 |
| **VITROS Anti-SARS-CoV-2 (Ortho Clinical Diagnostics)** | S/Co | Spike (S1) | 3 | J15 | 2.38 | 20-1887 | 2.73 |
| **VITROS Anti-SARS-CoV-2 (Ortho Clinical Diagnostics)** | S/Co | Spike (S1) | 3 | J12 | 2.64 | 20-1886 | 7.03 |
| **VITROS Anti-SARS-CoV-2 (Ortho Clinical Diagnostics)** | S/Co | Spike (S1) | 3 | J11 | 2.40 | 20-1885 | 6.56 |
| **VITROS Anti-SARS-CoV-2 (Ortho Clinical Diagnostics)** | S/Co | Spike (S1) | 3 | J1 | 3.08 | 20-1882 | 3.86 |
| **VITROS Anti-SARS-CoV-2 (Ortho Clinical Diagnostics)** | S/Co | Spike (S1) | 3 | D9 | 2.58 | 20-1879 | 41.70 |
| **VITROS Anti-SARS-CoV-2 (Ortho Clinical Diagnostics)** | S/Co | Spike (S1) | 3 | D17 | 2.67 | 20-1878 | 4.43 |
| **VITROS Anti-SARS-CoV-2 (Ortho Clinical Diagnostics)** | S/Co | Spike (S1) | 3 | D142 | 2.58 | 20-1877 | 3.68 |
| **VITROS Anti-SARS-CoV-2 (Ortho Clinical Diagnostics)** | S/Co | Spike (S1) | 4 | J7 | 2.75 | 20-1955 | 5.09 |
| **VITROS Anti-SARS-CoV-2 (Ortho Clinical Diagnostics)** | S/Co | Spike (S1) | 4 | J30 | 2.67 | 20-1954 | 3.68 |
| **VITROS Anti-SARS-CoV-2 (Ortho Clinical Diagnostics)** | S/Co | Spike (S1) | 4 | J19 | 2.86 | 20-1952 | 5.44 |
| **VITROS Anti-SARS-CoV-2 (Ortho Clinical Diagnostics)** | S/Co | Spike (S1) | 4 | J15 | 2.07 | 20-1887 | 2.75 |
| **VITROS Anti-SARS-CoV-2 (Ortho Clinical Diagnostics)** | S/Co | Spike (S1) | 4 | J12 | 2.28 | 20-1886 | 8.56 |
| **VITROS Anti-SARS-CoV-2 (Ortho Clinical Diagnostics)** | S/Co | Spike (S1) | 4 | J11 | 2.44 | 20-1885 | 8.08 |
| **VITROS Anti-SARS-CoV-2 (Ortho Clinical Diagnostics)** | S/Co | Spike (S1) | 4 | J1 | 3.13 | 20-1882 | 4.68 |
| **VITROS Anti-SARS-CoV-2 (Ortho Clinical Diagnostics)** | S/Co | Spike (S1) | 4 | D9 | 2.51 | 20-1879 | 60.80 |
| **VITROS Anti-SARS-CoV-2 (Ortho Clinical Diagnostics)** | S/Co | Spike (S1) | 4 | D17 | 2.75 | 20-1878 | 4.69 |
| **VITROS Anti-SARS-CoV-2 (Ortho Clinical Diagnostics)** | S/Co | Spike (S1) | 4 | D142 | 2.87 | 20-1877 | 4.08 |
| **Architect SARS-CoV-2 (Abbott)** | S/Co | Nucleocapsid | 2 | J7 | 0.03 | 20-1955 | 0.05 |
| **Architect SARS-CoV-2 (Abbott)** | S/Co | Nucleocapsid | 2 | J30 | 0.02 | 20-1954 | 0.08 |
| **Architect SARS-CoV-2 (Abbott)** | S/Co | Nucleocapsid | 2 | J19 | 0.03 | 20-1952 | 0.53 |
| **Architect SARS-CoV-2 (Abbott)** | S/Co | Nucleocapsid | 2 | J15 | 0.01 | 20-1887 | 0.28 |
| **Architect SARS-CoV-2 (Abbott)** | S/Co | Nucleocapsid | 2 | J12 | 0.03 | 20-1886 | 0.20 |
| **Architect SARS-CoV-2 (Abbott)** | S/Co | Nucleocapsid | 2 | J11 | 0.02 | 20-1885 | 1.52 |
| **Architect SARS-CoV-2 (Abbott)** | S/Co | Nucleocapsid | 2 | J1 | 0.01 | 20-1882 | 0.52 |
| **Architect SARS-CoV-2 (Abbott)** | S/Co | Nucleocapsid | 2 | D9 | 0.01 | 20-1879 | 0.59 |
| **Architect SARS-CoV-2 (Abbott)** | S/Co | Nucleocapsid | 2 | D17 | 0.02 | 20-1878 | 0.26 |
| **Architect SARS-CoV-2 (Abbott)** | S/Co | Nucleocapsid | 2 | D142 | 0.02 | 20-1877 | 1.02 |
| **Architect SARS-CoV-2 (Abbott)** | S/Co | Nucleocapsid | 3 | J7 | 0.04 | 20-1955 | 0.07 |
| **Architect SARS-CoV-2 (Abbott)** | S/Co | Nucleocapsid | 3 | J30 | 0.02 | 20-1954 | 0.11 |
| **Architect SARS-CoV-2 (Abbott)** | S/Co | Nucleocapsid | 3 | J19 | 0.04 | 20-1952 | 0.77 |
| **Architect SARS-CoV-2 (Abbott)** | S/Co | Nucleocapsid | 3 | J15 | 0.02 | 20-1887 | 0.34 |
| **Architect SARS-CoV-2 (Abbott)** | S/Co | Nucleocapsid | 3 | J12 | 0.04 | 20-1886 | 0.27 |
| **Architect SARS-CoV-2 (Abbott)** | S/Co | Nucleocapsid | 3 | J11 | 0.02 | 20-1885 | 1.96 |
| **Architect SARS-CoV-2 (Abbott)** | S/Co | Nucleocapsid | 3 | J1 | 0.02 | 20-1882 | 0.78 |
| **Architect SARS-CoV-2 (Abbott)** | S/Co | Nucleocapsid | 3 | D9 | 0.02 | 20-1879 | 0.81 |
| **Architect SARS-CoV-2 (Abbott)** | S/Co | Nucleocapsid | 3 | D17 | 0.02 | 20-1878 | 0.31 |
| **Architect SARS-CoV-2 (Abbott)** | S/Co | Nucleocapsid | 3 | D142 | 0.02 | 20-1877 | 1.36 |
| **Architect SARS-CoV-2 (Abbott)** | S/Co | Nucleocapsid | 4 | J7 | 0.04 | 20-1955 | 0.08 |
| **Architect SARS-CoV-2 (Abbott)** | S/Co | Nucleocapsid | 4 | J30 | 0.02 | 20-1954 | 0.14 |
| **Architect SARS-CoV-2 (Abbott)** | S/Co | Nucleocapsid | 4 | J19 | 0.04 | 20-1952 | 0.87 |
| **Architect SARS-CoV-2 (Abbott)** | S/Co | Nucleocapsid | 4 | J15 | 0.02 | 20-1887 | 0.47 |
| **Architect SARS-CoV-2 (Abbott)** | S/Co | Nucleocapsid | 4 | J12 | 0.04 | 20-1886 | 0.36 |
| **Architect SARS-CoV-2 (Abbott)** | S/Co | Nucleocapsid | 4 | J11 | 0.03 | 20-1885 | 2.40 |
| **Architect SARS-CoV-2 (Abbott)** | S/Co | Nucleocapsid | 4 | J1 | 0.02 | 20-1882 | 0.91 |
| **Architect SARS-CoV-2 (Abbott)** | S/Co | Nucleocapsid | 4 | D9 | 0.02 | 20-1879 | 0.96 |
| **Architect SARS-CoV-2 (Abbott)** | S/Co | Nucleocapsid | 4 | D17 | 0.03 | 20-1878 | 0.33 |
| **Architect SARS-CoV-2 (Abbott)** | S/Co | Nucleocapsid | 4 | D142 | 0.02 | 20-1877 | 1.66 |
| **GSP/DELFIA Anti-SARS-CoV-2 (PerkinElmer)** | S/Co | Spike (S1) | 2 | J7 | 0.42 | 20-1955 | 1.41 |
| **GSP/DELFIA Anti-SARS-CoV-2 (PerkinElmer)** | S/Co | Spike (S1) | 2 | J30 | 0.36 | 20-1954 | 2.02 |
| **GSP/DELFIA Anti-SARS-CoV-2 (PerkinElmer)** | S/Co | Spike (S1) | 2 | J19 | 0.55 | 20-1952 | 9.62 |
| **GSP/DELFIA Anti-SARS-CoV-2 (PerkinElmer)** | S/Co | Spike (S1) | 2 | J15 | 0.33 | 20-1887 | 3.37 |
| **GSP/DELFIA Anti-SARS-CoV-2 (PerkinElmer)** | S/Co | Spike (S1) | 2 | J12 | 0.72 | 20-1886 | 6.60 |
| **GSP/DELFIA Anti-SARS-CoV-2 (PerkinElmer)** | S/Co | Spike (S1) | 2 | J11 | 0.35 | 20-1885 | 16.37 |
| **GSP/DELFIA Anti-SARS-CoV-2 (PerkinElmer)** | S/Co | Spike (S1) | 2 | J1 | 0.41 | 20-1882 | 5.13 |
| **GSP/DELFIA Anti-SARS-CoV-2 (PerkinElmer)** | S/Co | Spike (S1) | 2 | D9 | 0.36 | 20-1879 | 75.08 |
| **GSP/DELFIA Anti-SARS-CoV-2 (PerkinElmer)** | S/Co | Spike (S1) | 2 | D17 | 0.27 | 20-1878 | 14.15 |
| **GSP/DELFIA Anti-SARS-CoV-2 (PerkinElmer)** | S/Co | Spike (S1) | 2 | D142 | 0.37 | 20-1877 | 9.75 |
| **In-house, University of Toronto** | S/Co | Spike | 2 | J7 | 0.41 | 20-1955 | 1.82 |
| **In-house, University of Toronto** | S/Co | Spike | 2 | J30 | 0.33 | 20-1954 | 2.13 |
| **In-house, University of Toronto** | S/Co | Spike | 2 | J19 | 0.73 | 20-1952 | 2.71 |
| **In-house, University of Toronto** | S/Co | Spike | 2 | J15 | 0.39 | 20-1887 | 2.21 |
| **In-house, University of Toronto** | S/Co | Spike | 2 | J12 | 0.50 | 20-1886 | 2.50 |
| **In-house, University of Toronto** | S/Co | Spike | 2 | J11 | 0.36 | 20-1885 | 2.88 |
| **In-house, University of Toronto** | S/Co | Spike | 2 | J1 | 0.31 | 20-1882 | 2.11 |
| **In-house, University of Toronto** | S/Co | Spike | 2 | D9 | 0.39 | 20-1879 | 2.73 |
| **In-house, University of Toronto** | S/Co | Spike | 2 | D17 | 0.21 | 20-1878 | 2.61 |
| **In-house, University of Toronto** | S/Co | Spike | 2 | D142 | 0.58 | 20-1877 | 2.61 |
| **In-house, University of Toronto** | S/Co | Spike (RBD) | 3 | J7 | 0.85 | 20-1955 | 1.31 |
| **In-house, University of Toronto** | S/Co | Spike (RBD) | 3 | J30 | 0.50 | 20-1954 | 1.64 |
| **In-house, University of Toronto** | S/Co | Spike (RBD) | 3 | J19 | 0.79 | 20-1952 | 4.08 |
| **In-house, University of Toronto** | S/Co | Spike (RBD) | 3 | J15 | 0.48 | 20-1887 | 2.48 |
| **In-house, University of Toronto** | S/Co | Spike (RBD) | 3 | J12 | 0.88 | 20-1886 | 3.25 |
| **In-house, University of Toronto** | S/Co | Spike (RBD) | 3 | J11 | 0.77 | 20-1885 | 3.89 |
| **In-house, University of Toronto** | S/Co | Spike (RBD) | 3 | J1 | 0.64 | 20-1882 | 2.77 |
| **In-house, University of Toronto** | S/Co | Spike (RBD) | 3 | D9 | 0.66 | 20-1879 | 6.08 |
| **In-house, University of Toronto** | S/Co | Spike (RBD) | 3 | D17 | 0.37 | 20-1878 | 4.36 |
| **In-house, University of Toronto** | S/Co | Spike (RBD) | 3 | D142 | 0.38 | 20-1877 | 4.27 |
| **In-house, University of Toronto** | S/Co | Nucleocapsid | 4 | J7 | 0.53 | 20-1955 | 0.46 |
| **In-house, University of Toronto** | S/Co | Nucleocapsid | 4 | J30 | 0.33 | 20-1954 | 0.50 |
| **In-house, University of Toronto** | S/Co | Nucleocapsid | 4 | J19 | 0.38 | 20-1952 | 0.95 |
| **In-house, University of Toronto** | S/Co | Nucleocapsid | 4 | J15 | 0.15 | 20-1887 | 0.79 |
| **In-house, University of Toronto** | S/Co | Nucleocapsid | 4 | J12 | 0.36 | 20-1886 | 1.21 |
| **In-house, University of Toronto** | S/Co | Nucleocapsid | 4 | J11 | 0.30 | 20-1885 | 0.96 |
| **In-house, University of Toronto** | S/Co | Nucleocapsid | 4 | J1 | 0.34 | 20-1882 | 0.97 |
| **In-house, University of Toronto** | S/Co | Nucleocapsid | 4 | D9 | 0.30 | 20-1879 | 1.23 |
| **In-house, University of Toronto** | S/Co | Nucleocapsid | 4 | D17 | 0.10 | 20-1878 | 1.37 |
| **In-house, University of Toronto** | S/Co | Nucleocapsid | 4 | D142 | 0.21 | 20-1877 | 1.56 |
| **In-house, University of Ottawa** | S/Co | Spike, polyclonal | 2 | J7 | 0.28 | 20-1955 | 2.02 |
| **In-house, University of Ottawa** | S/Co | Spike, polyclonal | 2 | J30 | 0.10 | 20-1954 | 2.85 |
| **In-house, University of Ottawa** | S/Co | Spike, polyclonal | 2 | J19 | 0.63 | 20-1952 | 7.49 |
| **In-house, University of Ottawa** | S/Co | Spike, polyclonal | 2 | J15 | 0.24 | 20-1887 | 3.39 |
| **In-house, University of Ottawa** | S/Co | Spike, polyclonal | 2 | J12 | 0.28 | 20-1886 | 6.96 |
| **In-house, University of Ottawa** | S/Co | Spike, polyclonal | 2 | J11 | 0.14 | 20-1885 | 4.00 |
| **In-house, University of Ottawa** | S/Co | Spike, polyclonal | 2 | J1 | 0.16 | 20-1882 | 4.00 |
| **In-house, University of Ottawa** | S/Co | Spike, polyclonal | 2 | D9 | 0.16 | 20-1879 | 4.00 |
| **In-house, University of Ottawa** | S/Co | Spike, polyclonal | 2 | D17 | 0.16 | 20-1878 | 6.40 |
| **In-house, University of Ottawa** | S/Co | Spike, polyclonal | 2 | D142 | 0.22 | 20-1877 | 7.87 |
| **In-house, University of Ottawa** | S/Co | Spike (RBD), polyclonal | 2 | J7 | 0.49 | 20-1955 | 1.27 |
| **In-house, University of Ottawa** | S/Co | Spike (RBD), polyclonal | 2 | J30 | 0.28 | 20-1954 | 1.43 |
| **In-house, University of Ottawa** | S/Co | Spike (RBD), polyclonal | 2 | J19 | 0.41 | 20-1952 | 6.14 |
| **In-house, University of Ottawa** | S/Co | Spike (RBD), polyclonal | 2 | J15 | 0.37 | 20-1887 | 2.63 |
| **In-house, University of Ottawa** | S/Co | Spike (RBD), polyclonal | 2 | J12 | 0.59 | 20-1886 | 4.48 |
| **In-house, University of Ottawa** | S/Co | Spike (RBD), polyclonal | 2 | J11 | 0.41 | 20-1885 | 5.22 |
| **In-house, University of Ottawa** | S/Co | Spike (RBD), polyclonal | 2 | J1 | 0.37 | 20-1882 | 3.58 |
| **In-house, University of Ottawa** | S/Co | Spike (RBD), polyclonal | 2 | D9 | 0.45 | 20-1879 | 4.00 |
| **In-house, University of Ottawa** | S/Co | Spike (RBD), polyclonal | 2 | D17 | 0.23 | 20-1878 | 7.14 |
| **In-house, University of Ottawa** | S/Co | Spike (RBD), polyclonal | 2 | D142 | 0.29 | 20-1877 | 7.67 |
| **In-house, University of Ottawa** | S/Co | Nucleocapsid, polyclonal | 2 | J7 | 0.36 | 20-1955 | 2.24 |
| **In-house, University of Ottawa** | S/Co | Nucleocapsid, polyclonal | 2 | J30 | 0.36 | 20-1954 | 1.79 |
| **In-house, University of Ottawa** | S/Co | Nucleocapsid, polyclonal | 2 | J19 | 0.27 | 20-1952 | 6.52 |
| **In-house, University of Ottawa** | S/Co | Nucleocapsid, polyclonal | 2 | J15 | 0.36 | 20-1887 | 5.49 |
| **In-house, University of Ottawa** | S/Co | Nucleocapsid, polyclonal | 2 | J12 | 0.50 | 20-1886 | 7.83 |
| **In-house, University of Ottawa** | S/Co | Nucleocapsid, polyclonal | 2 | J11 | 0.32 | 20-1885 | 4.00 |
| **In-house, University of Ottawa** | S/Co | Nucleocapsid, polyclonal | 2 | J1 | 0.80 | 20-1882 | 6.48 |
| **In-house, University of Ottawa** | S/Co | Nucleocapsid, polyclonal | 2 | D9 | 0.60 | 20-1879 | 8.30 |
| **In-house, University of Ottawa** | S/Co | Nucleocapsid, polyclonal | 2 | D17 | 0.09 | 20-1878 | 7.41 |
| **In-house, University of Ottawa** | S/Co | Nucleocapsid, polyclonal | 2 | D142 | 0.18 | 20-1877 | 8.67 |
| **In-house, University of Ottawa** | S/Co | Spike, monoclonal | 2 | J7 | 0.71 | 20-1955 | 2.18 |
| **In-house, University of Ottawa** | S/Co | Spike, monoclonal | 2 | J30 | 0.25 | 20-1954 | 2.25 |
| **In-house, University of Ottawa** | S/Co | Spike, monoclonal | 2 | J19 | 0.65 | 20-1952 | 2.56 |
| **In-house, University of Ottawa** | S/Co | Spike, monoclonal | 2 | J15 | 0.39 | 20-1887 | 2.44 |
| **In-house, University of Ottawa** | S/Co | Spike, monoclonal | 2 | J12 | 0.40 | 20-1886 | 2.77 |
| **In-house, University of Ottawa** | S/Co | Spike, monoclonal | 2 | J11 | 0.39 | 20-1885 | 3.00 |
| **In-house, University of Ottawa** | S/Co | Spike, monoclonal | 2 | J1 | 0.33 | 20-1882 | 2.47 |
| **In-house, University of Ottawa** | S/Co | Spike, monoclonal | 2 | D9 | 0.27 | 20-1879 | 4.00 |
| **In-house, University of Ottawa** | S/Co | Spike, monoclonal | 2 | D17 | 0.27 | 20-1878 | 2.98 |
| **In-house, University of Ottawa** | S/Co | Spike, monoclonal | 2 | D142 | 0.44 | 20-1877 | 2.73 |
| **In-house, University of Ottawa** | S/Co | Spike (RBD), monoclonal | 2 | J7 | 0.78 | 20-1955 | 1.92 |
| **In-house, University of Ottawa** | S/Co | Spike (RBD), monoclonal | 2 | J30 | 0.31 | 20-1954 | 2.16 |
| **In-house, University of Ottawa** | S/Co | Spike (RBD), monoclonal | 2 | J19 | 0.51 | 20-1952 | 4.63 |
| **In-house, University of Ottawa** | S/Co | Spike (RBD), monoclonal | 2 | J15 | 0.46 | 20-1887 | 3.61 |
| **In-house, University of Ottawa** | S/Co | Spike (RBD), monoclonal | 2 | J12 | 0.70 | 20-1886 | 4.63 |
| **In-house, University of Ottawa** | S/Co | Spike (RBD), monoclonal | 2 | J11 | 0.54 | 20-1885 | 5.23 |
| **In-house, University of Ottawa** | S/Co | Spike (RBD), monoclonal | 2 | J1 | 0.41 | 20-1882 | 3.97 |
| **In-house, University of Ottawa** | S/Co | Spike (RBD), monoclonal | 2 | D9 | 0.51 | 20-1879 | 4.00 |
| **In-house, University of Ottawa** | S/Co | Spike (RBD), monoclonal | 2 | D17 | 0.32 | 20-1878 | 4.00 |
| **In-house, University of Ottawa** | S/Co | Spike (RBD), monoclonal | 2 | D142 | 0.46 | 20-1877 | 5.22 |
| **In-house, University of Ottawa** | S/Co | Nucleocapsid, monoclonal | 2 | J7 | 0.39 | 20-1955 | 2.05 |
| **In-house, University of Ottawa** | S/Co | Nucleocapsid, monoclonal | 2 | J30 | 0.14 | 20-1954 | 1.74 |
| **In-house, University of Ottawa** | S/Co | Nucleocapsid, monoclonal | 2 | J19 | 0.20 | 20-1952 | 2.20 |
| **In-house, University of Ottawa** | S/Co | Nucleocapsid, monoclonal | 2 | J15 | 0.35 | 20-1887 | 4.00 |
| **In-house, University of Ottawa** | S/Co | Nucleocapsid, monoclonal | 2 | J12 | 0.47 | 20-1886 | 4.00 |
| **In-house, University of Ottawa** | S/Co | Nucleocapsid, monoclonal | 2 | J11 | 0.39 | 20-1885 | 4.00 |
| **In-house, University of Ottawa** | S/Co | Nucleocapsid, monoclonal | 2 | J1 | 0.78 | 20-1882 | 4.00 |
| **In-house, University of Ottawa** | S/Co | Nucleocapsid, monoclonal | 2 | D9 | 0.43 | 20-1879 | 4.00 |
| **In-house, University of Ottawa** | S/Co | Nucleocapsid, monoclonal | 2 | D17 | 0.14 | 20-1878 | 4.00 |
| **In-house, University of Ottawa** | S/Co | Nucleocapsid, monoclonal | 2 | D142 | 0.24 | 20-1877 | 4.00 |

DBS punches: 6 mm (1/4 inch) dried blood spot punch.
